# Interactions between gut microbiota and Parkinson's disease: The role of microbiota-derived amino acid metabolism

**DOI:** 10.3389/fnagi.2022.976316

**Published:** 2022-11-02

**Authors:** Wang Wang, Shujun Jiang, Chengcheng Xu, Lili Tang, Yan Liang, Yang Zhao, Guoxue Zhu

**Affiliations:** ^1^Department of Neurology, Nanjing Hospital of Chinese Medicine Affiliated to Nanjing University of Chinese Medicine, Nanjing University of Chinese Medicine, Nanjing, China; ^2^School of Medicine and Holistic Integrative Medicine, Nanjing University of Chinese Medicine, Nanjing, China; ^3^Chinese Medicine Modernization and Big Data Research Center, Nanjing Hospital of Chinese Medicine Affiliated to Nanjing University of Chinese Medicine, Nanjing University of Chinese Medicine, Nanjing, China

**Keywords:** Parkinson's disease, gut microbiota, amino acid metabolism, microbiota-host interaction, gut microbiota-brain axis

## Abstract

Non-motor symptoms (NMS) of Parkinson's disease (PD), such as constipation, sleep disorders, and olfactory deficits, may emerge up to 20 years earlier than motor symptoms. A series of evidence indicates that the pathology of PD may occur from the gastrointestinal tract to the brain. Numerous studies support that the gut microbiota communicates with the brain through the immune system, special amino acid metabolism, and the nervous system in PD. Recently, there is growing recognition that the gut microbiota plays a vital role in the modulation of multiple neurochemical pathways *via* the “gut microbiota-brain axis” (GMBA). Many gut microbiota metabolites, such as fatty acids, amino acids, and bile acids, convey signaling functions as they mediate the crosstalk between gut microbiota and host physiology. Amino acids' abundance and species alteration, including glutamate and tryptophan, may disturb the signaling transmission between nerve cells and disrupt the normal basal ganglia function in PD. Specific amino acids and their receptors are considered new potential targets for ameliorating PD. The present study aimed to systematically summarize all available evidence on the gut microbiota-derived amino acid metabolism alterations associated with PD.

## Introduction

**Figure 1:**
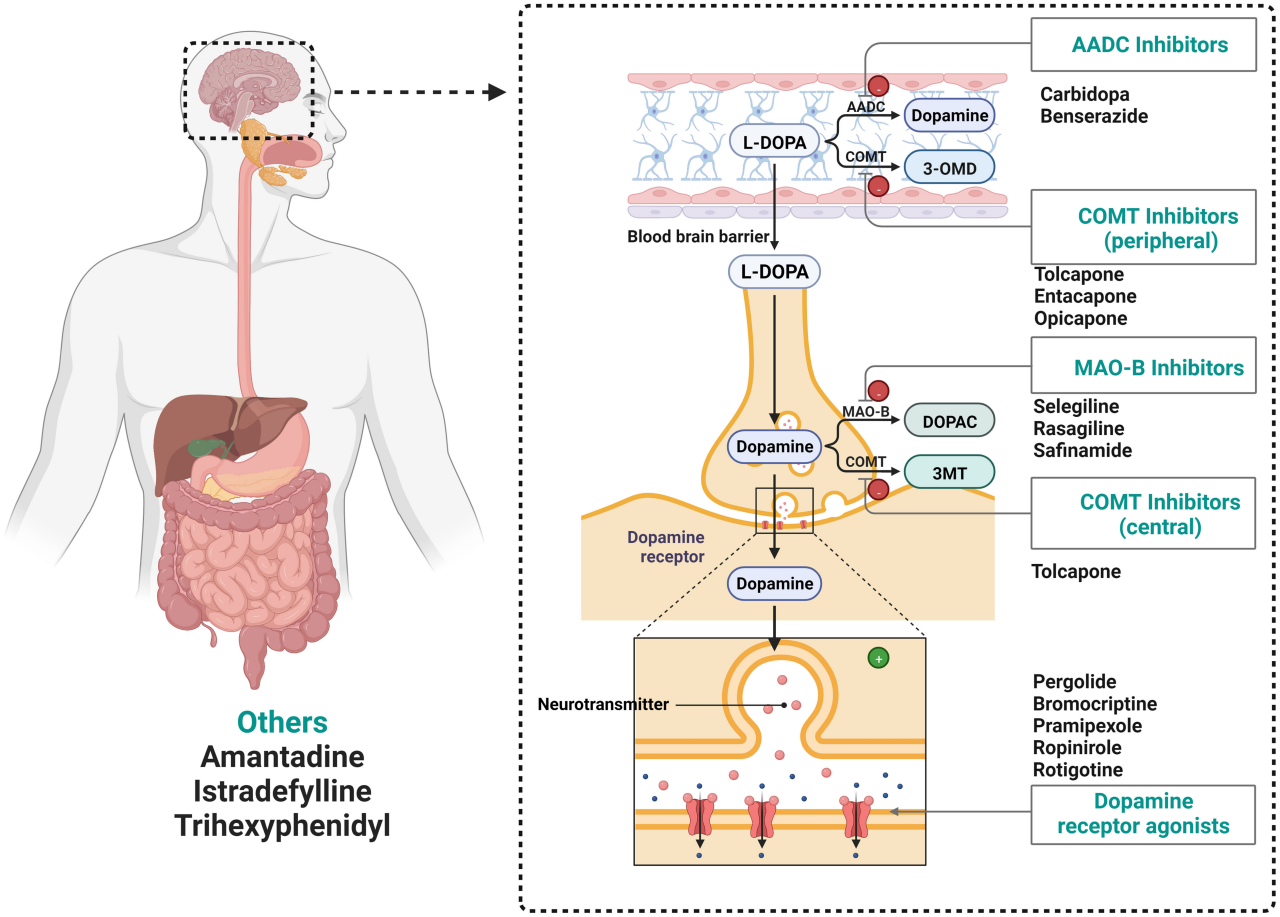
Drugs utilized in the treatment of Parkinson's disease. MAO-B, Monoamine oxidase-B inhibitors; COMT, catechol-O-methyl transferase inhibitors; L-DOPA, L-3,4-dihydroxyphenylalanine; AADC, amino acid decarboxylase (AADC, Dopa decarboxylase); DOPAC, 3,4-dihydroxyphenylacetic acid; 3-OMD, 3-O-methyl-dopa; 3-MT, 3-methoxytyramine.

Parkinson's disease (PD) is the second most common neurodegenerative disease and affects an estimated 3 million people worldwide. The typical feature of PD is the accumulation of intracellular aggregates called Lewy bodies, composed largely of the presynaptic protein alpha-synuclein (Collier et al., 2017). In clinical practice, individuals with PD benefit from drugs developed to act on a single molecular target (Figure 1; Sarkar et al., 2016; Tambasco et al., 2018). Levodopa (L-DOPA) is still the gold standard therapy for patients with PD. However, long-term L-DOPA treatment is associated with motor fluctuations and dyskinesias. As the disease progresses, amino acid decarboxylase inhibitors (AADC inhibitors), catechol-O-methyl transferase inhibitors (COMT inhibitors), monoamine oxidase B inhibitors (MAO-B inhibitors), and dopamine receptor agonists are also used. The surgical treatment of deep brain stimulation can be used for patients with PD who have severe limitations in functioning despite optimal pharmacological treatment (Weiner, 2002; Malek, 2019). However, there is not currently a medication available to cure or halt the progression of the disease.

The intestine is a complex and dynamic ecosystem caused by various microbial communities known as the gut microbiota (Zhao and Liu, 2020). The human intestinal tract contains more than 10-100 trillion microorganisms (Duttaroy, 2021; Zhang J. et al., 2021), which contribute to food digestion, nutrient provision, and combat pathogen invasion. Numerous studies have demonstrated that the gut microbiota plays a vital role in maintaining human health and adjusting numerous physiological functions, such as the nervous system, cardiovascular system, and immune system (Izco et al., 2021). Considering the importance of host-microbiota interactions for human health, access to yet unexplored resources will lead to the discovery of new actors regulating this crosstalk and therefore new potential therapeutic targets (Alam and Neish, 2018; Chang and Kao, 2019). Although genetic, societal, environmental, and other influencing factors are closely involved in disease etiology and progression, the host-microbiota interaction is increasingly recognized for its influence in a variety of diseases (Duttaroy, 2021), such as obesity (Arnoriaga-Rodríguez et al., 2020), inflammatory bowel disease (Sankarasubramanian et al., 2020), non-alcoholic fatty liver disease (Rom et al., 2020), and Parkinson's disease (Vascellari et al., 2020). More importantly, a better understanding of the host-microbiota interaction will improve an understanding of the relationship between gut microbiota and health. Interestingly, the change in microbial composition, intestinal dysfunction, bacterial metabolites, and endocrine function in the intestine is associated with PD (Sun et al., 2017). In addition, the abundance of *Prevotellaceae* presented a declining trend in PD patients compared with healthy individuals. Simultaneously, the abundance of *Enterobacteriaceae* has been found to be associated with the severity of postural instability and gait difficulty of PD patients (Scheperjans et al., 2015). Hence, the interaction between the gut microbiota and the occurrence of PD will provide a bright future for intervention, especially for the diagnosis and therapy of PD. Growing evidence suggests that the modulation of gut microbiota could be a novel therapeutic target in PD patients.

**Figure 2:**
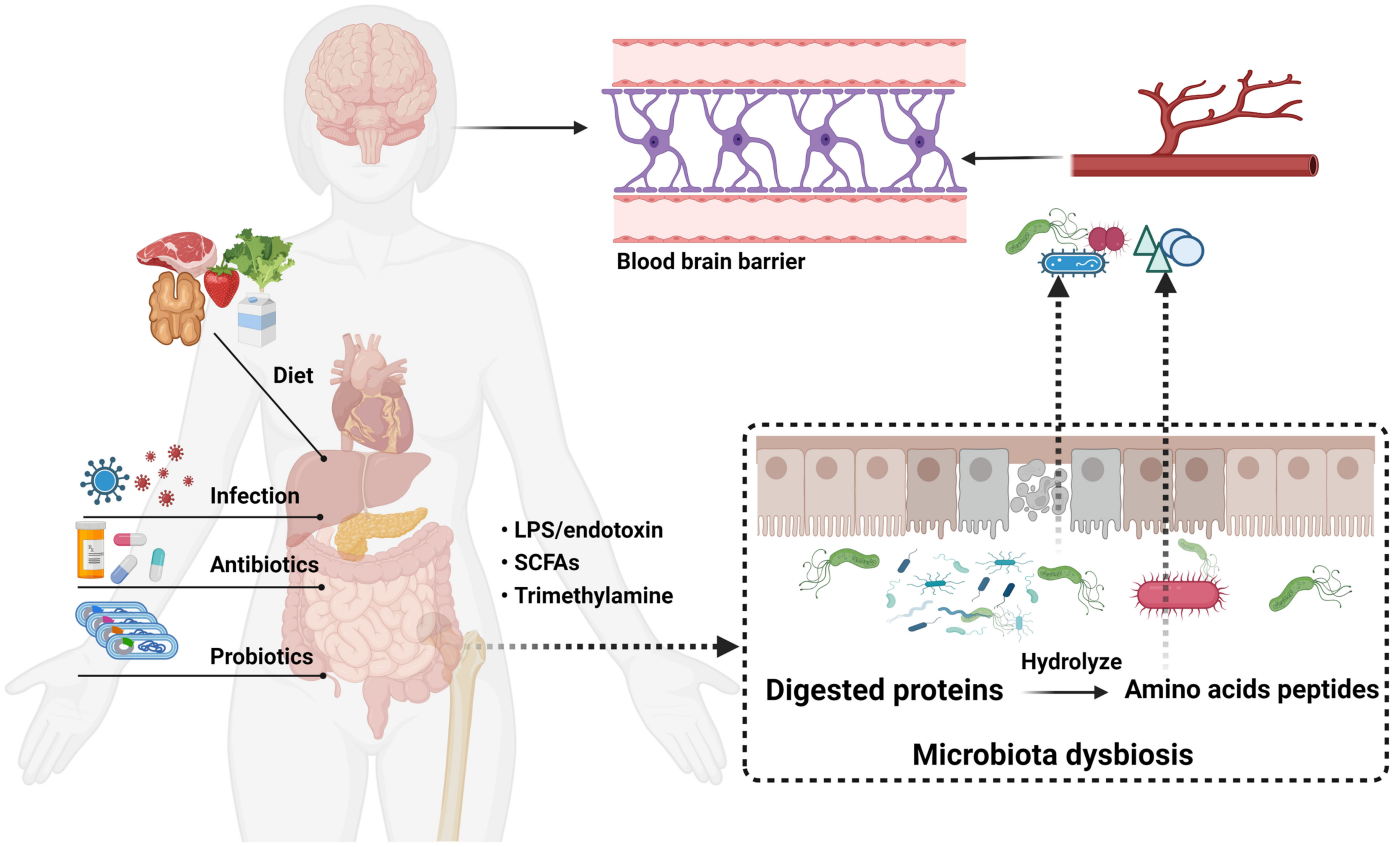
Microbiota-gut-brain axis communication pathway linking gut microbial dysbiosis with brain function in PD.

Amino acids are fundamental elements for protein and peptide synthesis. More importantly, a series of evidence indicates that amino acids are also important bioactive molecules that play key roles in signaling pathways and metabolic regulation (Sankarasubramanian et al., 2020). The gut microbiota composition related to protein metabolism in the intestinal tract is similar to that in feces and important in amino acid homeostasis and health (Bishu, 2016; Lin et al., 2017; Zhao et al., 2019). The result showed that the primary bacteria, such as *Klebsiella* spp., *Escherichia coli*, and *Anaerovibrio lipolytica*, are associated with protein metabolism. In addition, amino acids could be directly metabolized by some of these bacteria which own the ability to excrete numerous proteases and peptidases (Fan et al., 2017). Evidence shows that amino acids are exchanged between gut microbiota and the host (Metges, 2000). Thus, there is no doubt that a profound grasp of the significance of gut microbiota-derived amino acid metabolism has become imperative. Notwithstanding, the effects of amino acid metabolism on PD pathogenesis have not been comprehensively reviewed at present. In this review, detailed descriptions of the relationship between gut microbiota-derived amino acid metabolism and PD are summarized, and dysbiosis of microbial metabolite and microbiota-targeted interventions in PD disease are also discussed (Figure 2).

## Gut microbiota and Parkinson's disease

### The hypothesis of PD in the gut

Few researchers investigated the pathogenesis of PD prior to 1980. The mechanism remained elusive until the description of Lewy bodies first appeared. The main hypotheses involved in the pathogenesis of PD were as follows: oxidative stress (Trist et al., 2019), mitochondrial dysfunction (Navarro and Boveris, 2009), excitotoxicity (Ambrosi et al., 2014), inflammation, neurotrophic factor-deficiency (Lindahl et al., 2020), and the theory of gut origin (Postuma, 2015). Among them, oxidative stress and PD originating from the gut have attracted more attention. Accumulating evidence illustrated that oxidative stress may be the cause of many neurodegenerative diseases, including PD (Trist et al., 2019). Low levels of oxidative stress could promote mitochondria elimination and protect their biological function, while opposite effects appear in high-level oxidative stress beyond the cellular capacity by affecting the mitochondrial membrane's potential and protein synthesis in cytoplasm (Lee and Wei, 2005; Barodia et al., 2017). Previous studies mainly focused on the abnormal deposition of α-syn in the central nervous system in PD pathogenesis. Recently, multitudes of studies have elucidated that gut microbiota homeostasis and metabolites are correlated with the pathogenesis of PD. Simultaneously, Yang et al. (2019) revealed the existence of a bidirectional network called GMBA. The GMBA could affect human behavior and brain neurochemistry by regulating neurotransmitters and their receptors in PD patients. Taken altogether, the gut origin hypothesis implicates the gut as a potential origin of PD pathogenesis, offering fresh insights into the mechanisms underlying PD.

### A bidirectional link between intestinal disorders and neurodegeneration in the pathogenesis of Parkinson's disease

Relevant preclinical and epidemiological studies illustrated that the occurrence of common intestinal disorders, such as colorectal cancer (CRC), constipation, irritable bowel syndrome (IBS), inflammatory bowel disease (IBD), and small intestinal bacterial overgrowth syndrome (SIBO), were associated with PD by affecting the central nervous system (Barboza et al., 2015). Interestingly, a bidirectional link between the brain and gut plays an essential role in the association between intestinal dysfunction and PD. The association between PD and intestinal disorders is described in Table 1.

**Table 1:** The association between Parkinson's disease and intestinal disorders.

**Intestinal disorders**	**Connection with Parkinson's disease (PD)**	**Publication trends on web of science (accessed on 7 May 2022)**	**References**
Colorectal cancer (CRC)	1. PD patients had a reduced risk of CRC	“Parkinson's disease” and “Colorectal cancer” (176)	Xie et al., 2017; Fang et al., 2021
	2. CRC occurrence was significantly lower in patients with PD		
Constipation	1. Constipation patients are at a 2.27-fold increased risk of developing PD compared to the control group, and this phenomenon emerges up to 20 years before diagnosis	Parkinson's disease and “Constipation” (1,016)	Kaye et al., 2006; Adams-Carr et al., 2016; Svensson et al., 2016; Gan et al., 2018; Zhou et al., 2019; Camacho et al., 2021; Kang et al., 2022; Santos García et al., 2022; Zheng et al., 2022
	2. Compared with people without constipation, regional neural activity and functional connectivity in the brain show much difference in PD patients with constipation		
	3. Constipation is associated with a sustained increased risk of a PD diagnosis and progression of neurodegenerative pathology, and there was a higher incidence for men than women		
	4. Constipation is associated with cognitive decline in PD patients		
	5. Constipation is associated with the increased severity of motor symptoms and decreased dopamine levels in PD patients in a dose-dependent manner. Simultaneously, the different constipation-loading times could lead to different clinical characteristics, especially in motor symptoms		
Irritable bowel syndrome (IBS)	1. Patients with IBS are at an increased risk of developing PD in Taiwan in both genders in an age-dependent manner	“Parkinson's disease” and “Irritable bowel syndrome” (134)	Lai et al., 2014; Mertsalmi et al., 2017; Liu B. et al., 2021; Zhang J. et al., 2021; Lu et al., 2022; Yoon et al., 2022; Zhang X. et al., 2021
	2. IBS increased PD risk only in individuals ≥ 65 years		
	3. PD patients with IBS-like symptoms had more non-motor symptoms		
Inflammatory bowel disease (IBD)	1. The overall risk of PD in IBD, both Crohn's disease and ulcerative colitis is significantly higher than in controls	Parkinson's disease and “Inflammatory bowel disease” (302)	Lin et al., 2016; Killinger et al., 2018; Peter et al., 2018; Park et al., 2019; Villumsen et al., 2019; Zhu et al., 2019, 2022; Fu et al., 2020; Noh et al., 2020; Herrick and Tansey, 2021; Kim et al., 2022
	2. IBD is associated with increased PD risk regardless of sex, especially in patients over 65 years of age. Furthermore, the therapies for IBD using corticosteroids, anti-TNF and early anti-inflammatory methods may decrease the risk of PD		
	3. The risk of neurodegenerative diseases is higher in IBD patients than in the non-IBD population		
	4. Abnormal changes in the intestinal environment trigger the onset of PD *via* the brain-gut axis		
	5. Gut inflammation and higher LRRK2 levels in Crohn's disease (IBD) may be a biomarker of increased risk for sporadic PD		
Small intestinal bacterial overgrowth syndrome (SIBO)	1. The risk of SIBO is higher in PD patients than in non-PD patients, and SIBO could influence the progression of PD using negative and positive manners	“Parkinson's disease” and “Small intestinal bacterial overgrowth syndrome” (128)	Marrinan et al., 2014; Tan et al., 2014; Niu et al., 2016; Dǎnǎu et al., 2021
	2. SIBO may lead to fluctuation in the absorption of medications utilized to therapy PD, which could further influence the treatment of PD		
	3. SIBO is associated with increased motor fluctuations present in PD patients compared with individuals without SIBO		

PD and CRC are different diseases with diverse pathogenic mechanisms. Gut microbiota diversity (Xie et al., 2017), melatonin (Schernhammer et al., 2006), dopamine (Sarkar et al., 2015), and smoking (Hernán et al., 2002) may account for the bidirectional link between PD and CRC. The disorders of the ubiquitin-proteasome system may lead to the formation and accumulation of Lewy bodies including α-syn (Sherman and Goldberg, 2001). More importantly, the level of ubiquitin proteasome system is usually up-regulated in CRC patients (Manasanch and Orlowski, 2017). Notwithstanding, there is a clear need for more prospective studies to validate these hypotheses. Interestingly, phosphatidylinositol-3-kinase/protein kinase B/mammalian target of rapamycin (PI3K/Akt/mTOR) are over-expressed in CRCs (Bahrami et al., 2018), while the PI3K/Akt/mTOR activation could prevent PD *via* reducing the dopaminergic neuron apoptosis (Leikas et al., 2017).

Constipation, the second most common non-motor symptom of PD after anosmia, is characterized by infrequent stools, difficult stool passage, or both. Previous studies indicated that the main cause of constipation was slowed colonic transit. Furthermore, constipation is one of the indicators and occurs more than 20 years earlier than the diagnosis of PD (Frazzitta et al., 2019). Simultaneously, gastrointestinal dysfunction is a common feature of PD and could lead to the impaired absorption of L-DOPA, which also causes the motor fluctuations of PD (Svensson et al., 2016). Despite copious research, the exact mechanism of IBS remains unclear. Genetic variation, altered gut microbiota, increased gut permeability, and low-grade inflammation are risk factors for IBS (Ohman and Simrén, 2010). Compared with those without IBS, an individual with IBS is at a 48% increased risk of PD based on the meta-analysis and systematic review methods (Zhang J. et al., 2021). The risk factors of IBS can make the gastrointestinal tract more vulnerable to bacterial endotoxin and pathogens which may cause an increase in α-syn expression and aggregation (Mertsalmi et al., 2021). Furthermore, the previous research about the correlation between the gut microbiota and the occurrence and development of IBS has been observed and the gut-brain axis plays a vital role, which was consistent with the hypothesis of “PD originates in the gut” (Braak et al., 2006; Raskov et al., 2016; Canakis et al., 2020). The altered gut microbiota and increased gut permeability can activate systemic inflammation and enteric neuroglial cells, thereby initiating the development of α-syn pathology (Klingelhoefer and Reichmann, 2015). An increasing number of clinical studies revealed that IBD could cause neuroinflammation through the gut-brain axis which was consistent with the elevated stool calprotectin levels in PD patients (Schwiertz et al., 2018). Intestinal inflammation could accelerate the aggregation of α-syn in the gut and then spread to the brain, finally leading to PD (Stokholm et al., 2016).

SIBO is characterized by the excessive levels of bacteria colonized in the small intestine causing inflammation and malabsorption. The pathogenesis, clinical manifestation, and progression of sporadic PD might be affected by SIBO (Dobbs et al., 2012; DiBaise et al., 2018; Manole et al., 2021). Several studies found that H_2_-predominant vs. methane-predominant SIBO shows various effects on PD progression. In addition, because of different drug bioavailability and absorption, SIBO has conflicting effects on intestinal symptoms of PD with different pharmacological interventions (Gibson and Barrett, 2010; Maini Rekdal et al., 2019; van Kessel et al., 2019). Among the pathogenesis of PD, SIBO may cause local and systemic reactions that would further destroy the integrity of the intestinal barrier by influencing tight junctions and intestinal permeability. Many studies illustrated that the intestinal disorder may serve as a warning sign for PD.

The association pathogenesis of PD and gastrointestinal disorders was mainly focused on genetic factors, diet, environmental toxins, and gut microbiota, etc. Interestingly, PD is characterized by severe motor and NMS that can result in debilitating gastrointestinal (GI) symptoms. Specifically, the association between intestinal disorders and PD pathogenesis attracting many researchers' attention may be due to the existence of the gut-brain axis. Overall, the PD risk was higher in IBS patients than others, indicating that the intestinal disorder may serve as a warning sign for PD. Further research is warranted to explore the underlying mechanisms of this correlation.

### Roles of gut microbial dysbiosis and microbial products in PD

The key pathological characteristics of PD are the accumulation of α-syn and cell death in the brain's basal ganglia, affecting an estimated three million people (more than 60 years of age) (Wolters and Braak, 2006; Sulzer, 2007). This damage to dopaminergic neurons is responsible for the distinctive movement disorder and vagal nerve dysfunction associated with PD. Until recently, the NMS including constipation, dysphagia, disrupted sleep architecture, impaired olfaction, and depression were presented in PD patients (Raval et al., 2020). A great quantity of studies has shown that constipation is an early manifestation of the neurodegenerative process underlying PD. More importantly, the NMS of GI dysfunction (i.e., constipation) often appears much earlier in PD patients, predating the onset of motor symptoms by as many as 20 years (Abbott et al., 2001; Savica et al., 2009). Many studies found that gut microbiota composition alters in various PD-related NMS, possibly pre-dating motor symptoms (Heintz-Buschart et al., 2018). Interestingly, current research studies have illustrated that gut microbial disturbance can damage the intestinal barrier and raise chronic inflammation both in the gut (de La Serre et al., 2010; de Theije et al., 2014) and brain (Sampson and Mazmanian, 2015; Sampson et al., 2016).

#### The intestinal barrier

**Figure 3:**
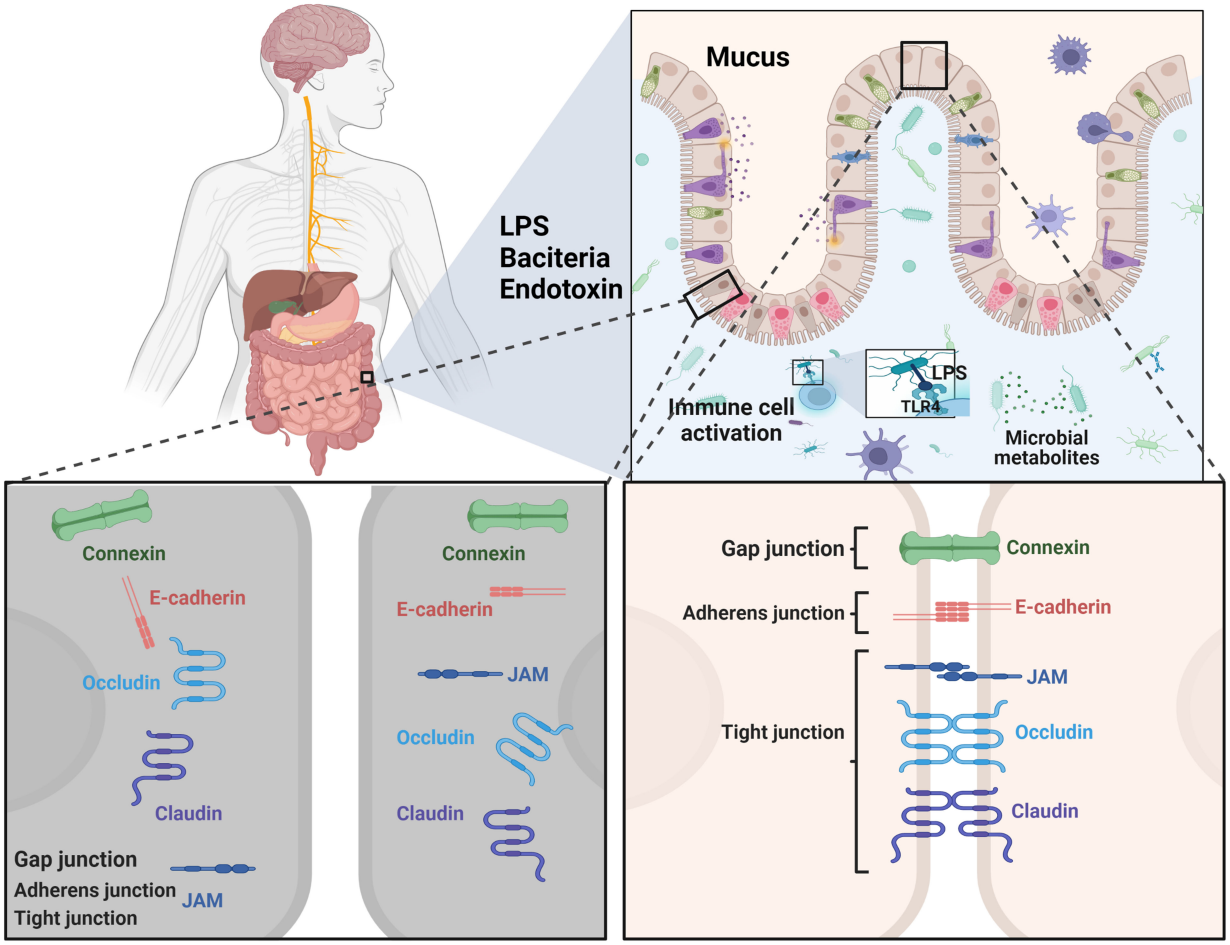
A schematic overview of the main cell types and physical defense mechanisms as targets related to intestinal barrier function for therapeutic strategies in PD.

The role of the intestinal barrier as an interface between the external and internal environments of the host is an obvious focus. The intestinal barrier facilitates the absorption of nutrients and prevents the entry of harmful intraluminal components into the blood circulation, such as endotoxins, and deleterious rhizobacteria (Furness et al., 1999; Sun and Shen, 2018). The tight junction complexes in epithelial cells, the mucosal surface, and the immune system are vital for the intact intestinal barrier and function, of which the first line of defense is the intestinal lumen itself (Figure 3; Schoultz and Keita, 2020; Gharib-Naseri et al., 2021).

Commensal luminal bacteria inhibit the colonization of pathogens by, for example, the production of bacteriocins, pH modification of the luminal content, and competition for nutrients required for the growth of pathogens (Bharadia et al., 2020). Otherwise, the commensal microbiota resides in the intestinal epithelium enhanced physiological paracellular permeability, and the mucus layer (Schoultz and Keita, 2019).

The single cell layer of epithelium consists of plentiful cell types and could divide the body and the outside luminal milieu. The tight barrier of the intestinal luminal milieu is established by a large quantity of cells and their functions (Schoultz and Keita, 2019). It is shown that antimicrobial peptides could be produced by Paneth cells by eliminating pathogenic bacteria (Wang et al., 2018). Simultaneously, innate and adaptive immune cells, such as neutrophils, T cells, monocytes, and natural killer cells, respond to the intrusion of xenobiotics and suppress the inflammation. Neutrophils, a kind of immunological cells, are among the first cells to reach inflamed areas and eliminate microorganisms through phagocytosis (Rosales et al., 2016). T-regulatory cells, a specific subpopulation of lymphocytes, are critical in the maintenance of immune homeostasis in suppressing the activation of various immune cells related to inflammation (Corthay, 2009). The crosstalk established between the gut microbiota and the intestinal epithelium is key to maintaining homeostasis in the gut. More importantly, the bi-directional communications between the innate immune system and pattern recognition receptors, such as Toll-like receptors (TLRs), scavenger receptors, and nucleotide-binding oligomerization domain (NOD)-like receptors (NLRs), are crucial to the intestinal barrier homeostasis in some conditions. The intestinal epithelial and immune cells are constantly in contact with foreign material and then respond appropriately to their intrusions, protecting the host (Wells et al., 2011). This interactivity ability makes the identification of external ingredients possible based on the antigen presenting cells. Even more interesting is that these cells can migrate to the peripheral site and transmit antigens to T-cells, leading to increased secretion of pro-inflammatory cytokines, IFN-γ for example, to improve the intestinal barrier (Wallace et al., 2014). Simultaneously, the enzyme of indoleamine 2,3-dooxygenase (Bessede et al., 2014), owning the effect of regulating the inflammatory reaction and the ability of protective effect against IBD, is responsible for the transformation of tryptophan to kynurenine (Arsenescu et al., 2011). TLRs are pro-inflammatory and conserved transmembrane receptors that can identify conserved molecular structures to detect microorganisms (Kubinak and Round, 2012). Pathogen triggered TLRs are generally understood to cause an inflammatory immune response, supporting the subsequent clearance of pathogenic microorganisms (Mayne et al., 2020). da Silva et al. (2016) found that these receptors were regulated by peripheral leukocyte responses in PD patients.

Intestinal permeability (between intestinal epithelial cells) is mainly characterized by medium-sized hydrophilic molecules transporting along their concentration gradient in a system without the assistance of a carrier system (Schoultz and Keita, 2020). A vital function of the intestinal epithelium is the maintenance of normal intestinal barrier function, which allows the selective permeability of essential nutrients, water, and ions without the entry of bacterial toxins and pathogens (Tabler et al., 2020). The increase in intestinal permeability represents intestinal barrier function disorders. A number of studies have found that increased permeability is correlated with the development of several diseases. Higher intestinal permeability induces the translocation of gut bacteria and microbial components that initiate inflammation and oxidative stress in the enteric nervous system, resulting in enteric α-synucleinopathy in PD (Bjarnason et al., 2004). Simultaneously, these findings also have important clinical applications. Barrier integrity in IBD patients, whose endoscopy findings are normal, may be detected using confocal laser endomicroscopy, which may predict the relapses (Galipeau and Verdu, 2016). The development of reliable and sensitive methods to measure intestinal permeability could contribute to identifying patients who are at high risk of suffering from PD and assist in the development of preventative therapies (Galipeau and Verdu, 2016). PD is characterized by an altered gut microbiota composition, the intestinal barrier, and the enteric neuroimmune system, therefore it is possible that intestinal barrier dysfunction may play a key role in PD development and/or progression. Meanwhile, the significant role of intestinal permeability has been highlighted in PD, which lays the groundwork for novel PD therapy targeting the restoration of the intestinal barrier.

#### Inflammation of the gut and neuroinflammation of the brain

The maturation and function of microglia are affected by the altered cell proportions, while immature phenotypes are based on the circulating levels of pro-inflammatory and anti-inflammatory cytokines contributing to host homeostasis (Erny et al., 2015). The astrocytes and microglia can produce pro-inflammatory cytokines *via* TLRs activation (Lucas and Maes, 2013). TLRs can recognize microbial-associated molecular patterns (MAMPs), such as lipopolysaccharides (LPS), and are activated by changes in the composition of gut microbiota and gut permeability (Bowman et al., 2003). LPS can activate immune cells (such as macrophages, neutrophils, and dendritic) and promote the inflammation and permeability of the gut (Rhee, 2014). In particular, the activated immune cells can release various pro-inflammatory cytokines, binding with receptors expressed in neurons and glial cells, and then spread to the brain *via* the blood-brain-barrier and can result in the neuroinflammation and death of neurons in the brain (Dantzer et al., 2000). The new research illustrated that gut leakiness and endotoxemia owing to the alteration of microbiota led to the co-occurrence of intestinal inflammation and neuroinflammation *via* the activation of the TLR4 (Alhasson et al., 2017).

## Amino acids and gut microbiota-brain axis in PD

### Source and composition of amino acid in dietary proteins

In the gastrointestinal tract, enterocyte peptidases further hydrolyze resultant peptide products into amino acids, dipeptides, and tripeptides for enterocyte transport into the portal venous system. At the same time, amino acids serve as substrates for protein synthesis that are utilized for energy production under extreme conditions, such as disease for example. Amino acids are classified as essential amino acids, non-essential amino acids, and conditionally essential amino acids based on the species, age, disease, and condition dependence (Thalacker-Mercer et al., 2020). Essential amino acids, including tryptophan, valine, lysine, leucine, isoleucine, phenylalanine, threonine, and methionine, must be provided by dietary protein (Church et al., 2020; Verzola et al., 2020) and non-essential amino acids can be synthesized through other pathways.

Recently, amino acids, including total amino acid and free amino acid, have been found to exist in mushrooms, corn grain, peanut, soybean, and white rice, etc. Research has found that the average total free amino acid content of the mushroom was 4.35% (Sun et al., 2017). The relative amount of total amino acid is 8.76% in coffee silverskin (Machado et al., 2020), 10.67% in corn grain, 29.29% in peanut, 9.08% potato, and 8.49% in white rice (Hou et al., 2019). It should be noted that amino acids are substrates in polyamine synthesis by numerous enzymes in humans and microbes (Krzystek-Korpacka et al., 2020).

### The absorption and metabolization of amino acids in the intestine

**Figure 4:**
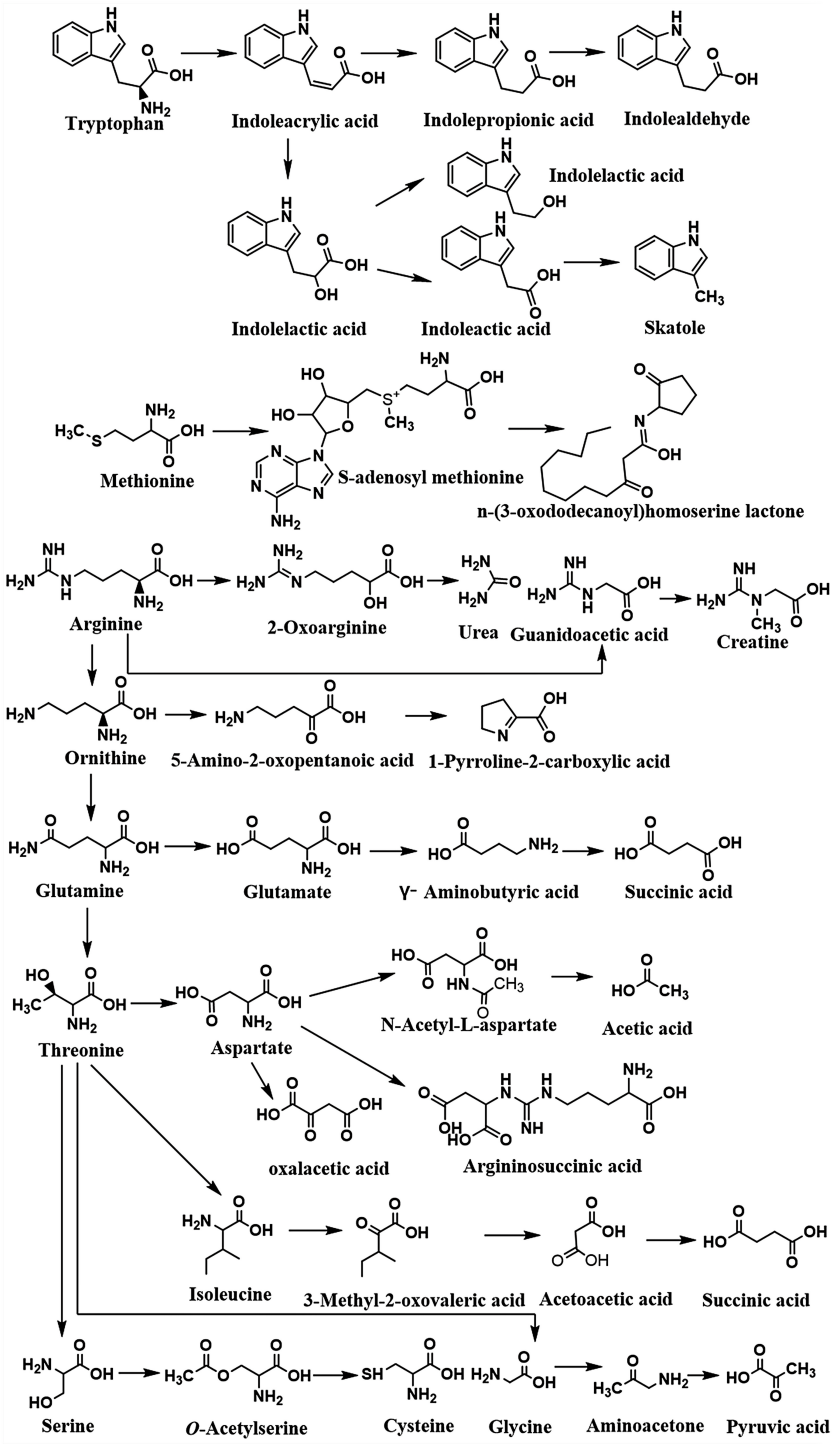
The summary of microbiota-derived amino acid metabolism profile.

Amino acids perform several crucial functions in the human body, either directly or indirectly. They are the main constituents of protein in bioactive substances and adjust the balance of energy and immunity in organisms. In addition, amino acids can be catalyzed through oxidized, reduced, fissioned, or coupled pathways (Davila et al., 2013). Several reactions including transamination, deamination, and decarboxylation are required steps in this process. Lots of work demonstrated that glycine, proline, and arginine usually act as the acceptors of hydrogen, and alanine, leucine, isoleucine, and histidine are hydrogen donors in the metabolic pathways (Rist et al., 2013). The metabolisms of relevant amino acids are summarized in Figure 4. The microorganisms are mixed with endogenous proteins, such as mucous proteins, chemicals secreted by the pancreas and gastric gland in the alimentary canal (Hayashi et al., 2005). The nitrogen-containing compounds are broken down into amino acids and peptides and related enzymes are excreted *via* the exocrine pancreas. The amino acids and peptides are absorbed by bacteria or spread from the intestinal lumen to the portal vein.

Amino acids are absorbed and digested in the intestinal epithelium (Bos et al., 2005; Hu and Guo, 2021). During the process, a tremendous amount of nitrogen-containing compounds passes by the ileocecal junction and gets into the posterior part of the intestine (Blachier et al., 2007). However, some proteins are still difficult to absorb and digest completely in the small intestinal. The remaining nitrogen-containing compounds without digestion are transported to the large intestine. Microorganisms play a vital role in protein hydrolysis to provide free amino acids for both host and microbial metabolism functions. Furthermore, most amino acids, synthesized by microorganisms, can be absorbed by the intestinal epithelial cells of the small and large intestines to implement a sequence of functions, like synthesis and catabolism (Laparra and Sanzm, 2010; Ma and Ma, 2019). The proteolysis and recycling of nitrogen-containing compounds are performed by proteolytic bacteria residing in the gastrointestinal tract (Macfarlane and Macfarlane, 2007). A large amount of amino acid maintains the immense and widespread metabolism via the function of microbiota. This diversity of amino acid metabolism in microorganisms may impart either active or passive effects on the host. Hence, adjusting protein or amino acid absorption and metabolization in the intestine may provide a strategy for shaping the amino acid fermenting bacteria and their metabolic pathways, thereby potentially affecting host metabolism.

### Amino acids metabolism and gut microbiota

The roles of gut microbiota on amino acid metabolism have been well investigated (Zhao et al., 2019). The great majority of digestive products, such as small peptides, amino acids, and tripeptides, are utilized by certain microbes or transported into systemic circulation by the small intestine. The functions of amino acids between the gut microbiota and host are bi-directional (Zhao et al., 2019). The gut microbiota plays an essential role in dietary protein and nitrogen recycling. In addition, the microbial activity induced by indigestible amino acids promotes the production of metabolic end products including short-chain fatty acids (SCFA; acetate, butyrate, propionate), branched-chain fatty acids, ammines, indoles, and ammonia, etc. (Macfarlane et al., 1988). Previous work revealed that bacterial metabolites appear to have beneficial or opposite effects on the host depending on their concentrations. As the major nutrients in the diet, amino acids are the basic components of proteins and peptides, and are essential for the emergence of organism-active molecules involved in the signaling pathways and metabolism modulation (Dai et al., 2015). Additionally, the gut microbiota is crucial for the metabolism and recycling of amino acids. The amino acids, deriving from the diet or the host, are utilized as the fundamental element by gut microbiota for the synthesis of proteins or produce a large number of metabolites by the nutritional metabolism, such as polyamines, phenolic, nitric oxide (NO), and hydrogen sulfide (H_2_S) (Dai et al., 2015). In addition, the “essential” amino acids, as a regulator for amino acid homeostasis in humans, can be synthesized by intestinal bacteria. For instance, research has illustrated that a large number of bacteria, like *Selenomonas ruminantium*, perform de novo synthesis of amino acids in the existence of peptides using experiments *in vitro* (Atasoglu et al., 1998; Neis et al., 2015). These results suggest that amino acid metabolism provides nutrition for gut microbiota and supports physiological action for the host (Dai et al., 2011; Collins et al., 2012). The intestinal bacterial communities are influenced by constituents of dietary protein, especially protein sources and concentrations. Proteins derived from plants are generally utilized in humans and animals. Nevertheless, crude proteins are characterized by low digestibility because of the anti-nutritional elements (He et al., 2015). By contrast, proteins originating from animals are characterized by high digestibility by aerobes in the large intestine. The homeostasis of gut microbiota (mainly in the large intestine) can be disorganized, leading to intestinal disorders (Fan et al., 2015). Consequently, it is essential to explore the connection between gut microbiota and amino acids (which are necessary for protein metabolism). In addition, comprehending the functions of different constituents of dietary protein on gut microbiota is crucial.

### Regulation of gut microbiota on amino acid metabolism and synthesis

An enormous number of metabolic pathways, such as glucose, lipid, and amino acid metabolism, are regulated by gut microbiota. The composition of gut microbiota-derived metabolites in the intestinal tract is very complex. The nutrition can be provided by amino acids acquired in the diet or synthesized by the host for the gut microbiota for protein production. Amino acids can be integrated into bacterial cells as amino acid residues in proteins, and further incorporated into the metabolic pathway. Amino acids and peptides are obtained by the hydrolysis of proteins through proteases and peptidases secreted by gut microbiota. Ultimately, amino acids and peptides could be absorbed by microbiota through specific transporters. However, the small bioactive molecules will encounter various consequences under different physiological conditions of humans (Davila et al., 2013). Transamination or deamination is the first stage of amino acid catabolism, followed by oxidation or reduction. Biogenic amines are alkaline organic compounds mainly produced by decarboxylation reactions of free amino acids present by the enzyme decarboxylase (Wu et al., 2021).

Recent studies demonstrated convincingly that a large number of amino acid-fermenting bacteria are inhabited the intestinal tract (up to 1,011 per gram of dry feces) (Atasoglu et al., 1998). The “essential” amino acid of lysine, produced by bacteria, is absorbed and integrated as a whole host protein *in vivo* studies (Metges et al., 1996, 1999). The research illustrated that the 15N-lysine originated from microbial lysine with the comparison of the integration of ^15^N from ^15^NH^4^Cl into the inside body system (Torrallardona et al., 1996; Gill et al., 2006). Additionally, gut microbiota-derived lysine and threonine were shown to be crucial for free lysine and threonine in a study on nitrogen adequate diets in humans (Metges et al., 1999). Furthermore, this research found that the abundance of gut microbiota was associated with the precursors generated by humans related to “essential” amino acids (Metges et al., 1999).

A genome-wide analysis illustrates that a number of *clostridium* species, *Clostridium acetobutylicum* for example, have comprehensive genes for the biosynthesis of amino acids, whereas *Clostridium perfringens* lack the genes for amino acid biosynthesis such as lysine, serine, glutamic acid, and aromatic amino acids (Kevin et al., 2016). Hence, in order to elucidate the functions of amino acids from the perspective of bacterial species, it is necessary to understand the biosynthesis of amino acids in microorganisms. However, nitrogen and amino acids, required for the growth and fermentation of glycolytic bacteria, could be provided by the residual dietary proteins in the large intestine (Mafra et al., 2013). Various *Lactobacillus* and *Bifidobacterium* strains can produce γ-aminobutyric acid which is a neural active substance (Barrett et al., 2012). It is acknowledged that imidazole propionate can be transformed into histidine-derived metabolite by gut microbiota (Koh et al., 2018). Moreover, gut microbiota can produce tryptophan owing to the existing of tryptophan decarboxylase in the intestinal tract (van de Wouw et al., 2017). Given the above, amino acids can be utilized for the synthesis of bacterial cell components and catabolized. Major amino acid-fermenting bacteria in the intestinal tract are concluded in Table 2.

**Table 2:** Major amino acid-fermenting bacteria in the digestive tract.

**Amino acids**	**Intestinal bacteria**	**Metabolite**	**Related mechanisms in PD**	**Trends in PD**	**Niches**	**References**
Phenylalanine	*Subdoligranulum*	Phenylpropionate	Phenylalanine is the precursor of dopamine and could participate in protein sequences in all tissues	Ascend	Rumen	Hirayama et al., 2016; Dodd et al., 2017
	*Lactobacillus*	Phenyl lactate			Small intestine	
	Clostridium	Phenylacetate			Cecum	
	*Pepto streptococcus* spp.	Phenyl pyruvic acid			Colon	
		4-OH-phenylpyruvic acid				
		Ammonia				
		CO_2_				
Asparagine	*Fusobacterium nucleatum*	Aspartic acid	Related to oxidative stress and dopamine cell degeneration in PD	Descend	Small intestine	Nagaraja et al., 2021
	*Escherichia coli*	Ammonia			Large intestine	
	*Klebsiella pneumoniae*	CO_2_			Colon	
	*Campylobacter jejuni*				Feces	
	*Bacteroides fragilis*					
Tryptophan	*Lactobacillus* spp.	Indole	Associated with psychiatric problems in advanced PD	Descend	Stomach	Hatano et al., 2016; Dehhaghi et al., 2019; Liu B. et al., 2021; Liu J. R. et al., 2021
	*Bifidobacterium* spp.	Indole ethanol			Small intestine	
	*Clostridium* spp.	Indolepropionic acid			Colon	
	*Pepto streptococcus* spp.	Indolelactic acid				
	*Bacteroides* spp.	Indoleacetic acid			Feces	
	*Clostridium sporogenes*	Skatole				
	*Ruminococcus gnavus*	Tryptamine				
		Indolealdehyde				
		Indoleacrylic acid				
Lysine	*Clostridium sticklandii*	Lysine-5,6-aminomutase	Acylation of lysine neutralizes may change the conformation of proteins	Ascend	Stomach	Potrykus et al., 2008; Wan et al., 2019
					Small intestine	
	*Porphyromonas gingivalis*	Lysine			Colon	
					Feces	
		2,3-aminomutase				
		2,5-diaminohexanoate				
		Dehydrogenase				
		3,5-diaminohexanoate				
		Dehydrogenase				
		3,6-diaminohexanoic acid				
Serine	Fusobacterium	Acetate	Signaling molecule to regulate the growth, repair, and maintenance of brain functions	Ascend	Stomach	Campbell et al., 1967; Schierack et al., 2007; Donatti et al., 2020
	Varium	Lactate			Small intestine	
	*Campylobacter jejuni*	Pyruvate			Colon	
	*Bacteroides fragilis*	Ammonia				
	*Acidaminococcus fermentans*	Malate			Feces	
		CO_2_			Rumen	
	*Clostridium aminophilum*				Cecum	
	*Clostridium perfringens*				Stomach	
	*Clostridium sticklandii*				Large intestine	
	*Pepto streptococcus* spp.					
Aspartic acid	*Fusobacterium nucleatum*	Asparagine	Related to oxidative stress, and dopamine cell degeneration in PD	Descend	Small intestine	Sugihara et al., 1974; Guccione et al., 2008
	*Escherichia coli*	Fumarate			Colon	
		Oxaloacetate				
	*Campylobacter jejuni*				Feces	
	*Acidaminococcus*					
	*Fermentans*					
	*Bacteroides fragilis*					
Glutamine	*Clostridium aminophilum*	Pyroglutamate	Involved in the glutamate-glutamine cycle	Ascend	Mouth	Donatti et al., 2020; Kumari et al., 2020
	*Selenomonas ruminantium*	Acetate			Small intestine	
	*Acidaminococcus fermentans*	Butyrate			Large intestine	
		Ammonia			Stomach	
	*Clostridium perfringens*	CO_2_				
					Colon	
	*Pepto streptococcus* spp.				Feces	
	*Streptococcus bovis*				Rumen	
	*Campylobacter jejuni*				Cecum	
	*Klebsiella pneumoniae*					
	*Escherichia coli*					
	*Fusobacterium nucleatum*					
Glutamate	*Clostridium aminophilum*	2-Oxaloacetate	Participating in the disruption of the normal basal ganglia function, thus leading to neuronal death	Descend	Mouth	Donatti et al., 2020; Jiménez-Jiménez et al., 2020; Kumari et al., 2020
	*Selenomonas ruminantium*	Ammonia			Small intestine	
	*Acidaminococcus fermentans*	GABA			Large intestine	
		CO_2_				
	*Clostridium perfringens*				Stomach	
	*Campylobacter jejuni*				Colon	
					Feces	
	*Klebsiella pneumoniae*				Cecum	
	*Escherichia coli*					
	*Fusobacterium varium*					
	*Fusobacterium nucleatum*					
Glutamic acid	*Fusobacterium nucleatum*	Glutamate	Glutamic acid is a precursor of glutathione and may reflect an increase in oxidative stress in the disease progression	Descend	Small intestine	Rychlik and Russell, 2002; Whitehead and Cotta, 2004; Anderson et al., 2009; Vascellari et al., 2020
	*Fusobacterium varium*	Ammonia			Large intestine	
		Acetate				
	*Escherichia coli*	Butyrate			Stomach	
	*Selenomonas ruminantium*	GABA				
	*Acidaminococcus fermentans*	CO_2_				
		2-Oxaloacetate				
	*Clostridium aminophilum*	2-ketoglutarate				
Histidine	*Fusobacterium nucleatum*	Urocanate	Suppressive neurotransmitter effects and hormone secretion	Ascend	Mouth	Attwood et al., 1998; Kumari et al., 2020; Sylte et al., 2020
					Small intestine	
	*Fusobacterium varium*	Ammonia			Large intestine	
	*Klebsiella pneumoniae*	Glutamic acid				
		Histamine			Colon	
		CO_2_			Feces	
					Rumen	
Glycine	*Fusobacterium nucleatum*	Acetate	Glycine could stimulate the release of dopamine and acetylcholine	Ascend	Mouth	Luan et al., 2015; Kumari et al., 2020; Sánchez-Andrea et al., 2020
	*Clostridium perfringens*	Pyruvate			Small intestine	
	*Bacteroides fragilis*	Serine			Large intestine	
					Stomach	
	*Escherichia coli*				Colon	
					Feces	
Threonine	*Fusobacterium nucleatum*	Acetate	Signaling molecule to regulate the growth, repair, and maintenance of brain functions	Ascend	Mouth	Donatti et al., 2020; Wu and Deng, 2020; Karkache et al., 2021
	*Escherichia coli*	Ammonia			Small intestine	
	*Pepto streptococcus* spp.	Propionate			Large intestine	
	*Clostridium sporogenes*	n-butyrate				
	*Clostridium sticklandii*	Ketobutyrate			Stomach	
	*Clostridium difficile*	Lactate			Colon	
	*Clostridium perfringens*	CO_2_			Feces	
	*Megasphaera elsdenii*					
	*Acidaminococcus fermentans*					
	*Bacteroides fragilis*					
Arginine	*Fusobacterium nucleatum*	2-Oxoarginine	Serve as a helpful clinical diagnostic biomarker for PD	Ascend	Mouth	Amano et al., 2020; Donatti et al., 2020; Martí I Líndez and Reith, 2021
	*Escherichia coli*	Guanidinoacetic acid			Small intestine	
	*Klebsiella pneumoniae*	Creatine			Large intestine	
	*Clostridium sticklandii*	Urea			Stomach	
	*Clostridium perfringens*	Putrescine			Colon	
	*Selenomonas ruminantium*	Spermine			Feces	
		Agmatine			Cecum	
		Ammonia			Rumen	
		CO_2_				
Alanine	*Staphylococcus aureus*	Isovalerate	Alanine may point to mitochondrial dysfunction, oxidative stress, and inflammation markers in PD	Ascend	Small intestine	Nielsen et al., 2020
	*Streptococcus* spp.	Isocaproate			Large intestine	
		Ammonia			Feces	
	Gram negative bacteria					
	*Escherichia coli*					
	*Selenomonas ruminantium*					
	*Prevotella* spp.					
	*Bacteroides* spp.					
	*Clostridium* spp.					
	Ruminal bacteria					
Valine	*Staphylococcus aureus*	Ammonia	Related to myelination dysfunction of the neurons.	Descend	Large intestine	Toczylowska et al., 2020
	*Streptococcus* spp.	CO_2_				
		Acetate				
	*Escherichia coli*	Isobutyrate				
	*Klebsiella* spp.					
	*Selenomonas ruminantium*					
	*Megasphaera elsdenii*					
Methionine	*Klebsiella pneumoniae*	S-adenosyl-L-methionine	Associated with hyperhomocysteinemia	Ascend	Small intestine	Toczylowska et al., 2020
		n-(3-oxododecanoyl) homoserine lactone			Colon	
					Feces	
Leucine	*Pepto streptococcus* spp.	Isovalerate	Contribute to muscle wasting, twitching, and tremors.	Descend	Rumen	Luan et al., 2015; Ma et al., 2020; Vascellari et al., 2020
		Isocaproate			Small intestine	
	*Clostridium bifermentans*	Ammonia			Cecum	
		CO2				
	*Clostridium sporogenes*	Acetate			Colon	
		Isobutyrate			Large intestine	
	*Clostridium sticklandii*					
	*Clostridium difficile*					
	*Prosthecobacter*					
Isoleucine	*Pepto streptococcus* spp.	Isovalerate	Contribute to muscle wasting, twitching, and tremors	Descend	Rumen	Kumari et al., 2020; Vascellari et al., 2020
	*Clostridium bifermentans*	Isocaproate			Small intestine	
	*Clostridium sporogenes*	Ammonia			Cecum	
	*Clostridium sticklandii*	CO_2_				
		Acetate			Colon	
	*Clostridium difficile*	Isobutyrate			Large intestine	
Linoleic acid	Bacteroidaceae	9-,13-oxoODEs	Associated with protective effects and may reflect an excess of oxidative stress	Descend	Small intestine	Vascellari et al., 2020
	*Streptomyces griseorubens*	Ammonia			Colon	
		CO_2_				
		Acetate			Feces	
Tyrosine	*Clostridium*	Ferulic acid	Tyrosine is then further hydroxylated to produce Dopa by tyrosine hydroxylase	Ascend	Stomach	Kumari et al., 2020
	*Bacteroides*	4-Hydroxycinnamic acid			Small intestine	
	*Bifidobacterium*	4-Hydroxyphenylacetic acid			Colon	
		4-Hydroxyphenylpropionic acid			Feces	
	*Faecalibacterium*	3-Hydroxybenzoic acid				
Pyruvate	*Faecalibacterium*	Acetate	Reduced pyruvate may be related to impairment in energetic and repair functions	Descend	Rumen	Toczylowska et al., 2020; Shen et al., 2021
	*Clostridium bifermentans*	Formate			Small intestine	
	*Clostridium sporogenes*	Lactate			Large intestine	
		Lactoyl-CoA				
		Acryloyl-CoA			Cecum	
		Propionyl-CoA			Colon	
		Propionate				

### Amino acids as precursors for microbially derived short-chain fatty acids

The literature reports that short-chain fatty acids (SCFAs) constitute a major class of fermentation products. The microbiota-derived carbohydrates of SCFAs are cardinal examples of beneficial metabolites (Sonnenburg and Sonnenburg, 2014). Acetate (accounts for ~50–70%), propionate (produced by the species of *Phylum Firmicutes*), and butyrate (produced by a small quantity of *Phylum Firmicutes*, such as *Faecalibacterium prausnitzii* and *Roseburia hominis*) (Sokol et al., 2008; Machiels et al., 2014) are SCFAs. These SCFAs supply key metabolic signaling that underlies homeostasis and the function of colonic Tregs by different molecular mechanisms (Mu et al., 2017; Yang, 2022). In faith, a wide variety of amino acids generated from microbial protein fermentation in the large intestine can be presented as precursors for short-chain fatty acid synthesis (Ciarlo et al., 2016). The great majority of amino acids such as glutamate, ornithine, and glycine can be metabolized by anaerobic bacteria and utilized for the synthesis of butyrate (Neis et al., 2015). Zou et al. (2021) reported that phenylalanine, tyrosine, and tryptophan could be metabolized to indolic and phenolic compounds *via* the gut microbiota. However, indolic and phenolic compounds can be utilized to mitigate inflammatory responses. Concurrently, it plays a significant role to promote the comprehension of the pathophysiology of diseases.

SCFAs have many ameliorative effects on gut health. First, SCFAs can maintain the integrity of the intestinal barrier and exert therapeutic potential for intestinal inflammation (Lewis et al., 2010). For instance, butyrate can activate adenosine monophosphate kinase (AMPK) or down-regulate the expression of claudin 2 to regulate the expression of tight junction proteins, eventually enhancing intestinal barrier function (Daly and Shirazi-Beechey, 2006; Peng et al., 2009). Second, SCFAs contribute to the mucous production in the gastrointestinal tract (Dalile et al., 2019). The research (Gaudier et al., 2009) illustrated that butyrate regulated MUC2 gene expression to regulate the production of mucin. Additionally, SCFAs can affect gastrointestinal motility by inhibiting histone deacetylases (Cherbut et al., 1998). Ultimately, SCFAs can interact with vagal afferents by influencing inflammation and hormonal regulation (Dalile et al., 2019).

To summarize, amino acids can alter the gut microbiota composition by forming an intestinal microenvironment favorable to the survival and proliferation of certain microbes (Libao-Mercado et al., 2009). Conversely, gut microbiota delivers energy and reduces co-factors *via* providing amino acids to meet the needs of the host (Metges, 2000). Gut microbiota owns the substitutable role (both the host nutrition and physiology) in amino acid metabolism, and the phenomenon shows the bidirectional process between amino acids and gut microbiota (Macfarlane and Macfarlane, 2012). Briefly, SCFAs play numerous biological roles: (1) constitute an energy source for muscles; (2) implicate in the transportation and metabolism of epithelial cells; and (3) influence epithelial cell growth and differentiation.

### Amino acid transporters in the large intestine

It is widely known that several amino acids are likely absorbed in the large intestine. The significance of the balance between whole-body proteins and amino acids is still unclear, especially in humans. The amino acids and peptide transporters in the intestinal tract contribute to amino acid absorption and metabolism in the large intestine (Hendriks et al., 2012).

A tremendous amount of solute carrier (SLC) transporters, which contain a large number of families of distinct transporters, are adopted to transport amino acids and peptides (including di- and tripeptides). Many above-mentioned transporters are expressed in the large intestine. There is evidence for transporter expression at different levels, such as the protein level and the gene expression level. Peptide transporter 1 (PEPT1), encoded by SLC family 15 member 1 (SLC15A1), is involved in the intestinal uptake of oligopeptides and peptide-mimetic drugs (Liu et al., 2019). As we have seen previously, SLC15A1 is the unique transporter that has been verified at the protein level in the human large intestine. The host relies on the system L transporters to obtain essential amino acids, such as SLC7A5, also known as L-type amino acid transporter (LAT)1, SLC7A8, also known as LAT2, SLC43A1, also known as LAT3, and SLC43A2, also known as LAT4. Both SLC7A5 and SLC7A82 are amino acid exchangers, while SLC43A1 and SLC43A2 facilitate amino acid proliferation (Wang and Holst, 2015). Some of the amino acids, such as tryptophan, tyrosine, isoleucine, leucine, valine, and phenylalanine, can be transported into cells via LAT1 and LAT2. Meanwhile, the other amino acids, such as Leucine, isoleucine, valine, phenylalanine, and methionine can be transported into cells by LAT3 and LAT4 (Wang and Holst, 2015). Furthermore, SLC6A14, coupled with a Na^+^ gradient, a Cl^−^ gradient, and membrane potential, shows higher expression in the colon than in the ileum and presents special characteristics with much a better sense of amino acids than the other three transporters (Anderson et al., 2009). Simultaneously, SLC36A1, also called proton-assisted amino acid transporter 1 (PAT1), shows a similar ability to SLC6A14. In addition, other transporter genes expressed in the large intestine discussed in current research also attract our attention. Furthermore, the SLC transporter functions of amino acids and peptides in the large intestine require further study.

### Amino acids regulate the intestinal epithelial barrier functions

The elementary mechanism of intestinal homeostasis relies on complex molecular crosstalk between host and gut microbiota. It is demonstrated that the catabolites of tryptophan by bacteria are various, such as tryptamine, skatole, indoleacrylic acid, indoleacetic acid, indolelactic acid, indolepropionic acid, and indolealdehyde, which are the ligands of the aryl hydrocarbon receptor. A recent study indicated that the catabolite of indole could improve intestinal epithelial barrier functions by regulating certain gene expressions (Roager and Licht, 2018). Simultaneously, the catabolite of indolepropionic acid acted as a ligand of the pregnane X receptor and showed the effect of adjusting the intestinal barrier function at the existence of indoles (Venkatesh et al., 2014). In addition, catabolite indoleacrylic acid was found to promote goblet cell differentiation and mucus production to regulate intestinal barrier function. In summary, the catabolites of tryptophan have the effect on improving intestinal barrier function by regulating the pregnane X receptor and aryl hydrocarbon receptor. The study demonstrated that glutamine improved IL-13-induced barrier dysfunction by up-regulating the expression of the tight junction protein claudin-1, through preventing the PI3K-Akt signaling pathway (Li et al., 2021), and consequently damaged the intestinal barrier function and increased intestinal permeability. At the same time, glutamate can up-regulate the expression of tight junction proteins and protect the diquat-induced oxidative stress to promote intestinal epithelial cell growth and health based on the new biochemical.

### The role of amino acids in Parkinson's disease

There is increasing evidence that dopamine dysfunction plays an important role in the pathogenesis of PD. There is also increasing evidence to suggest that the other amino acids, such as glutamate, γ-aminobutyric acid, homocysteine, and large neutral amino acids in the brain were involved in the pathogenesis of PD (Yuan et al., 2013; Figura et al., 2018). According to the research, dopamine treatment is associated with elevated homocysteine in Parkinson's disease (Kuhn et al., 1998). The three amino acids of glutamate, cysteine, and glycine are involved in the synthesis of glutathione. The ratio of the two forms of glutathione (reduced and oxidized forms) can affect the cellular redox status. Mitochondrial complex I has been considered central to the pathogenesis of PD. However, low glutathione concentrations may inhibit mitochondrial complex I (Müller et al., 2016). Meanwhile, the finding suggests that branched-chain amino acids (leucine, isoleucine, and valine) can serve as nitrogen donors to maintain the balance of glutamate-glutamine in astrocytes and neurons (Zhang et al., 2022). Branched-chain amino acids could promote the catabolism of glutamate which may exert beneficial effects on PD. Interestingly, tyrosine and phenylalanine are the key substrates for the production of dopamine in PD. The elevation of phenylacetyl-L-glutamine was positively correlated with firmer stool and constipation severity among PD patients. Simultaneously, gut microbiota-derived tryptophan catabolites could adjust the inflammatory response *via* affecting pro-inflammatory cytokines and lipogenesis in macrophages and hepatocytes (Shao et al., 2021). To compound the issue, glutamic acid and γ-aminobutyric acid are related to the clinical heterogeneity of PD, the symptoms of Parkinson's disease, and especially to the parkinsonian tremor (Yuan et al., 2013). Promisingly, the detection of amino acid neurotransmitter levels may offer new insight into the pathogenesis and early diagnosis of Parkinson's disease. Therefore, more stringent studies with a larger sample size are needed to explore the changes in amino acid neurotransmitter levels during Parkinson's disease progression.

## Conclusions

Researchers have found that amino acids provide powerful support for human health. In recent years, the gut microbiota has received great attention, putting it in the spotlight of biomedical research. The balance of intestinal microbes and the host play a vital role in several disorders, including the impairment of the nervous system. Notably, gut microbiota, a major determinant of bidirectional communication between the gut and brain, could be regulated by a number of mechanisms, and it is illustrated that the microbiota and its produced biochemical messengers are major facets of maintaining a balanced intestinal microenvironment. Microbiota-derived amino acid molecules contribute to host health through modulating immune function, gastrointestinal physiology function, and microbiota composition. Striatal dopamine depletion in PD contributes to the hyperactivity of subthalamic nucleus output pathways, resulting in an abnormal increase in specific microbiota-derived amino acid molecules (glutamate for example), which are involved in the degeneration of the nigrostriatal system based on the excitotoxicity mechanisms. All the studies have shown that GMBA may be significantly correlated with PD pathogenesis.

Although microbiota-host interaction has drawn much attention both from clinicians and researchers in recent years, it still calls for a deeper understanding of the complex GMBA communication. Furthermore, microbiota-derived amino acid metabolism is a crucial factor suggesting major perturbations of the microbiome. These studies illustrated that amino acids metabolized by gut microbiota might affect the degeneration of the nigrostriatal system in PD patients. Gut microbiota and amino acids may be potential therapeutic targets for PD.

## Author contributions

GZ and YZ contributed to the conceptualization. WW and GZ contributed to the investigation and funding. WW, GZ, SJ, CX, YL, and LT contributed to the writing and final approval. All authors contributed to the article and approved the submitted version.

## Funding

This work was funded by the National Natural Science Foundation of China (82204375 and 82003933), the Science and Technology Development Planning Project of Traditional Chinese Medicine of Jiangsu Province of China (QN202103), the Natural Science Foundation of Nanjing University of Chinese Medicine (XZR2021046), the Nanjing Medical Science and Technology Development Project (YKK20167), and Nanjing Youth Talent Training Plan of TCM (ZYQ20006).

## Conflict of interest

The authors declare that the research was conducted in the absence of any commercial or financial relationships that could be construed as a potential conflict of interest.

## Publisher's note

All claims expressed in this article are solely those of the authors and do not necessarily represent those of their affiliated organizations, or those of the publisher, the editors and the reviewers. Any product that may be evaluated in this article, or claim that may be made by its manufacturer, is not guaranteed or endorsed by the publisher.

## References

[B1] Abbott R. D. Petrovitch H. White L. R. Masaki K. H. Tanner C. M. Curb J. D.. (2001). Frequency of bowel movements and the future risk of Parkinson's disease. Neurology 57, 456–462. 10.1212/wnl.57.3.456 11502913

[B2] Adams-Carr K. L. Bestwick J. P. Shribman S. Lees A. Schrag A. Noyce A. J. (2016). Constipation preceding Parkinson's disease: a systematic review and meta-analysis. J. Neurol. Neurosurg. Psychiatry 87, 710–716. 10.1136/jnnp-2015-311680 26345189

[B3] Alam A. Neish A. (2018). Role of gut microbiota in intestinal wound healing and barrier function. Tissue Barriers 6, 1539595. 10.1212/WNL.57.3.456 30404570 PMC6389125

[B4] Alhasson F. Das S. Seth R. Dattaroy D. Chandrashekaran V. Ryan C. N.. (2017). Altered gut microbiome in a mouse model of Gulf War Illness causes neuroinflammation and intestinal injury via leaky gut and TLR4 activation. PLoS ONE 12, e0172914. 10.1371/journal.pone.0172914 28328972 PMC5362211

[B5] Amano G. Matsuzaki S. Mori Y. Miyoshi K. Han S. Shikada S. (2020). SCYL1 arginine methylation by PRMT1 is essential for neurite outgrowth via Golgi morphogenesis. Mol. Biol. Cell 31, 1963–1973. 10.1091/mbc.E20-02-0100 32583741 PMC7543066

[B6] Ambrosi G. Cerri S. Blandini F. (2014). A further update on the role of excitotoxicity in the pathogenesis of Parkinson's disease. J. Neural Transm. (Vienna) 121, 849–859. 10.1007/s00702-013-1149-z 24380931

[B7] Anderson C. M. Howard A. Walters J. R. Ganapathy V. Thwaites D. T. (2009). Taurine uptake across the human intestinal brush-border membrane is via two transporters: H+-coupled PAT1 (SLC36A1) and Na+- and Cl(–)-dependent TauT (SLC6A6). J. Physiol. 587, 731–744. 10.1113/jphysiol.2008.164228 19074966 PMC2669967

[B8] Arnoriaga-Rodríguez M. Mayneris-Perxachs J. Burokas A. Contreras-Rodríguez O. Blasco G. Coll C.. (2020). Obesity impairs short-term and working memory through gut microbial metabolism of aromatic amino acids. Cell Metab. 32, 548–560.e7. 10.1016/j.cmet.2020.09.002 33027674

[B9] Arsenescu R. Arsenescu V. Zhong J. Nasser M. Melinte R. Dingle R. W.. (2011). Role of the xenobiotic receptor in inflammatory bowel disease. Inflamm Bowel Dis. 17, 1149–1162. 10.1002/ibd.21463 20878756 PMC3013235

[B10] Atasoglu C. Valdés C. Walker N. D. Newbold C. J. Wallace R. J. (1998). De novo synthesis of amino acids by the ruminal bacteria *Prevotella bryantii* B14, *Selenomonas ruminantium* HD4, and *Streptococcus bovis* ES1. Appl Environ Microbiol. 64, 2836–2843. 10.1128/AEM.64.8.2836-2843.1998 9687438 PMC106780

[B11] Attwood G. T. Klieve A. V. Ouwerkerk D. Patel B. K. (1998). Ammonia-hyperproducing bacteria from New Zealand ruminants. Appl. Environ. Microbiol. 64, 1796–1804. 10.1128/AEM.64.5.1796-1804.1998 9572953 PMC106232

[B12] Bahrami A. Khazaei M. Hasanzadeh M. ShahidSales S. Joudi Mashhad M. Farazestanian M.. (2018). Therapeutic potential of targeting PI3K/AKT pathway in treatment of colorectal cancer: rational and progress. J. Cell Biochem. 119, 2460–2469. 10.1002/jcb.25950 28230287

[B13] Barboza J. L. Okun M. S. Moshiree B. (2015). The treatment of gastroparesis, constipation and small intestinal bacterial overgrowth syndrome in patients with Parkinson's disease. Exp. Opin. Pharmacother. 16, 2449–2464. 10.1517/14656566.2015.1086747 26374094

[B14] Barodia S. K. Creed R. B. Goldberg M. S. (2017). Parkin and PINK1 functions in oxidative stress and neurodegeneration. Brain Res. Bull. 133, 51–59. 10.1016/j.brainresbull.2016.12.004 28017782 PMC5718625

[B15] Barrett E. Ross R. P. O'Toole P. W. Fitzgerald G. F. Stanton C. (2012). γ-Aminobutyric acid production by culturable bacteria from the human intestine. J. Appl. Microbiol. 113, 411–417. 10.1111/j.1365-2672.2012.05344.x 22612585

[B16] Bessede A. Gargaro M. Pallotta M.T. Matino D. Servillo G. Brunacci C.. (2014). Aryl hydrocarbon receptor control of a disease tolerance defence pathway. Nature. 511, 184–190. 10.1038/nature13323 24930766 PMC4098076

[B17] Bharadia L. Agrawal N. Joshi N. (2020). Development and functions of the infant gut microflora: Western vs. Indian infants. Int. J. Pediatr. 2020, 7586264. 10.1155/2020/7586264 32454840 PMC7229554

[B18] Bishu S. (2016). Sensing of nutrients and microbes in the gut. Curr. Opin. Gastroenterol. 32, 86–95. 10.1097/MOG.0000000000000246 26836123

[B19] Bjarnason I. Takeuchi K. Bjarnason A. Adler S. N. Teahon K. (2004). The G.U.T. of gut. Scand. J. Gastroenterol. 39, 807–815. 10.1080/00365520410003326 15513377

[B20] Blachier F. Mariotti F. Huneau J. F. Tomé D. (2007). Effects of amino acid-derived luminal metabolites on the colonic epithelium and physiopathological consequences. Amino Acids 33, 547–562. 10.1007/s00726-006-0477-9 17146590

[B21] Bos C. Juillet B. Fouilletm H. Turlanm L. Daré S. Luengo C.. (2005). Postprandial metabolic utilization of wheat protein in humans. Am. J. Clin. Nutr. 81, 87–94. 10.1093/ajcn/81.1.87 15640465

[B22] Bowman C. C. Rasley A. Tranguch S. L. Marriott I. (2003). Cultured astrocytes express toll-like receptors for bacterial products. Glia 43, 281–291. 10.1002/glia.10256 12898707

[B23] Braak H. de Vos RA. Bohl J. Del Tredici K. (2006). Gastric alpha-synuclein immunoreactive inclusions in Meissner's and Auerbach's plexuses in cases staged for Parkinson's disease-related brain pathology. Neurosci Lett. 396, 67–72. 10.1016/j.neulet.2005.11.012 16330147

[B24] Camacho M. Macleod A. D. Maple-Grødem J. Evans J. R. Breen D. P. Cummins G.. (2021). Early constipation predicts faster dementia onset in Parkinson's disease. NPJ Parkinson's Dis. 7, 45. 10.1038/s41531-021-00191-w 34039994 PMC8154963

[B25] Campbell H. A. Mashburn L. T. Boyse E. A. Old L. J. (1967). Two L-asparaginases from *Escherichia coli* B. Their separation, purification, and antitumor activity. Biochemistry 6, 721–730. 10.1021/bi00855a011 5337885

[B26] Canakis A. Haroon M. Weber H. C. (2020). Irritable bowel syndrome and gut microbiota. Curr. Opin. Endocrinol. Diab. Obes. 27, 28–35. 10.1097/MED.0000000000000523 31789724

[B27] Chang C. S. Kao C. Y. (2019). Current understanding of the gut microbiota shaping mechanisms. J. Biomed. Sci. 26, 59. 10.1186/s12929-019-0554-5 31434568 PMC6702754

[B28] Cherbut C. Ferrier L. Rozé C. Anini Y. Blottière H. Lecannu G.. (1998). Short-chain fatty acids modify colonic motility through nerves and polypeptide YY release in the rat. Am. J. Physiol. 275, G1415–G1422. 10.1152/ajpgi.1998.275.6.G1415 9843779

[B29] Church D. D. Hirsch K. R. Park S. Kim I. Y. Gwin J. A. Pasiakos S. M.. (2020). Essential amino acids and protein synthesis: insights into maximizing the muscle and whole-body response to feeding. Nutrients. 12, 3717. 10.3390/nu12123717 33276485 PMC7760188

[B30] Ciarlo E. Heinonen T. Herderscheem J, Fenwickm, C, Mombelli, M. Le Roy D.. (2016). Impact of the microbial derived short chain fatty acid propionate on host susceptibility to bacterial and fungal infections in vivo. Sci. Rep. 6, 37944. 10.1038/srep37944 27897220 PMC5126587

[B31] Collier T. J. Kanaan N. M. Kordower J. H. (2017). Aging and Parkinson's disease: different sides of the same coin? Mov. Disord. 32, 983–990. 10.1002/mds.27037 28520211 PMC5844262

[B32] Collins S. M. Surette M. Bercik P. (2012). The interplay between the intestinal microbiota and the brain. Nat. Rev. Microbiol. 10, 735–742. 10.1038/nrmicro2876 23000955

[B33] Corthay A. (2009). How do regulatory T cells work? Scand. J. Immunol. 70, 326–336. 10.1111/j.1365-3083.2009.02308.x 19751267 PMC2784904

[B34] da Silva D. J. Borges A. F. Souza P. O. de Souza P. R. Cardoso C. R. Dorta M. L.. (2016). Decreased toll-like receptor 2 and toll-like receptor 7/8-induced cytokines in Parkinson's disease patients. Neuroimmunomodulation 23, 58–66. 10.1159/000443238 26886382

[B35] Dai Z. Wu Z. Hang S. Zhu W. Wu G. (2015). Amino acid metabolism in intestinal bacteria and its potential implications for mammalian reproduction. Mol. Hum. Reprod. 21, 389–409. 10.1093/molehr/gav003 25609213

[B36] Dai Z. L. Wu G. Zhu W. Y. (2011). Amino acid metabolism in intestinal bacteria: links between gut ecology and host health. Front. Biosci. (Landmark Ed). 16, 1768–1786. 10.2741/3820 21196263

[B37] Dalile B. Van Oudenhove L. Vervliet B. Verbeke K. (2019). The role of short-chain fatty acids in microbiota-gut-brain communication. Nat. Rev. Gastroenterol. Hepatol. 16, 461–478. 10.1038/s41575-019-0157-3 31123355

[B38] Daly K. Shirazi-Beechey S. P. (2006). Microarray analysis of butyrate regulated genes in colonic epithelial cells. DNA Cell Biol. 25, 49–62. 10.1089/dna.2006.25.49 16405400

[B39] Dǎnǎu A. Dumitrescu L. Lefter A. Tulbă D. Popescu B. O. (2021). Small intestinal bacterial overgrowth as potential therapeutic target in Parkinson's disease. Int. J. Mol. Sci. 22, 11663. 10.3390/ijms222111663 34769091 PMC8584211

[B40] Dantzer R. Konsman J. P. Bluthé R. M. Kelley K. W. (2000). Neural and humoral pathways of communication from the immune system to the brain: parallel or convergent? Auton. Neurosci. 85, 60–65. 10.1016/S1566-0702(00)00220-4 11189027

[B41] Davila A. M. Blachier F. Gotteland M. Andriamihaja M. Benetti P. H. Sanz Y.. (2013). Intestinal luminal nitrogen metabolism: role of the gut microbiota and consequences for the host. Pharmacol. Res. 68, 95–107. 10.1016/j.phrs.2012.11.005 23183532

[B42] de La Serre C. B. Ellis C. L. Lee J. Hartman A. L. Rutledge J. C. Raybould H. E. (2010). Propensity to high-fat diet-induced obesity in rats is associated with changes in the gut microbiota and gut inflammation. Am. J. Physiol. Gastrointest. Liver Physiol. 299, G440–G448. 10.1152/ajpgi.00098.2010 20508158 PMC2928532

[B43] de Theije C. G. Wopereis H. Ramadan M. van Eijndthoven T. Lambert J. Knol J.. (2014). Altered gut microbiota and activity in a murine model of autism spectrum disorders. Brain Behav. Immun. 37, 197–206. 10.1016/j.bbi.2013.12.005 24333160

[B44] Dehhaghi M. Kazemi Shariat Panahi H. Guillemin G. J. (2019). Microorganisms, tryptophan metabolism, and kynurenine pathway: a complex interconnected loop influencing human health status. Int. J. Tryptoph. Res. IJTR 12, 1178646919852996. 10.1177/1178646919852996 31258331 PMC6585246

[B45] DiBaise J. K. Crowell M. D. Driver-Dunckley E. Mehta S. H. Hoffman-Snyder C. Lin T.. (2018). Weight loss in Parkinson's disease: no evidence for role of small intestinal bacterial overgrowth. J Parkinsons Dis. 8, 571–581. 10.3233/JPD-181386 30149465

[B46] Dobbs R. J. Charlett A. Dobbs S. M. Weller C. A. Ibrahim M. A. Iguodala O.. (2012). Leukocyte-subset counts in idiopathic parkinsonism provide clues to a pathogenic pathway involving small intestinal bacterial overgrowth. A surveillance study. Gut Pathog. 4, 12. 10.1186/1757-4749-4-12 23083400 PMC3500215

[B47] Dodd D. Spitzer M. H. Van Treuren W. Merrill B. D. Hryckowian A. J. Higginbottom S. K.. (2017). A gut bacterial pathway metabolizes aromatic amino acids into nine circulating metabolites. Nature 551, 648–652. 10.1038/nature24661 29168502 PMC5850949

[B48] Donatti A. Canto A. M. Godoi A. B. da Rosa D. C. Lopes-Cendes I. (2020). Circulating metabolites as potential biomarkers for neurological disorders-metabolites in neurological disorders. Metabolites 10, 389. 10.3390/metabo10100389 33003305 PMC7601919

[B49] Duttaroy A. K. (2021). Role of gut microbiota and their metabolites on atherosclerosis, hypertension and human blood platelet function: a review. Nutrients. 13, 144. 10.3390/nu13010144 33401598 PMC7824497

[B50] Erny D. Hrabě de Angelis A. L. Jaitin D. Wieghofer P. Staszewski O. David E.. (2015). Host microbiota constantly control maturation and function of microglia in the CNS. Nat. Neurosci. 18, 965–977. 10.1038/nn.4030 26030851 PMC5528863

[B51] Fan P. Li L. Rezaei A. Eslamfam S. Che D. Ma X. (2015). Metabolites of dietary protein and peptides by intestinal microbes and their impacts on gut. Curr. Protein Pept. Sci. 16, 646–654. 10.2174/1389203716666150630133657 26122784

[B52] Fan P. Liu P. Song P. Chen X. Ma X. (2017). Moderate dietary protein restriction alters the composition of gut microbiota and improves ileal barrier function in adult pig model. Sci. Rep. 7, 43412. 10.1038/srep43412 28252026 PMC5333114

[B53] Fang H. Du Y. Pan S. Zhong M. Tang J. (2021). Patients with Parkinson's disease predict a lower incidence of colorectal cancer. BMC Geriatr. 21, 564. 10.1186/s12877-021-02497-z 34663210 PMC8522030

[B54] Figura M. Kuśmierska K. Bucior E. Szlufik S. Koziorowski D. Jamrozik Z.. (2018). Serum amino acid profile in patients with Parkinson's disease. PLoS One 13, e0191670. 10.1371/journal.pone.0191670 29377959 PMC5788376

[B55] Frazzitta G. Ferrazzoli D. Folini A. Palamara G. Maestri R. (2019). Severe constipation in Parkinson's disease and in parkinsonisms: prevalence and affecting factors. Front. Neurol. 10, 621. 10.3389/fneur.2019.00621 31275225 PMC6591373

[B56] Fu P. Gao M. Yung K. (2020). Association of intestinal disorders with parkinson's disease and alzheimer's disease: a systematic review and meta-analysis. ACS Chem. Neurosci. 11, 395–405. 10.1021/acschemneuro.9b00607 31876406

[B57] Furness J. B. Kunze W. A. Clerc N. (1999). Nutrient tasting and signaling mechanisms in the gut. II. The intestine as a sensory organ: neural, endocrine, and immune responses. Am. J. Physiol. 277, G922–G928. 10.1152/ajpgi.1999.277.5.G922 10564096

[B58] Galipeau H. J. Verdu E. F. (2016). The complex task of measuring intestinal permeability in basic and clinical science. Neurogastroenterol. Motil. 28, 957-65. 10.1111/nmo.12871 27339216

[B59] Gan J. Wan Y. Shi J. Zhou M. Lou Z. Liu Z. (2018). A survey of subjective constipation in Parkinson's disease patients in shanghai and literature review. BMC Neurol. 18, 29. 10.1186/s12883-018-1034-3 29544459 PMC5856226

[B60] Gaudier E. Rival M. Buisine M. P. Robineau I. Hoebler C. (2009). Butyrate enemas upregulate Muc genes expression but decrease adherent mucus thickness in mice colon. Physiol. Res. 58, 111–119. 10.33549/physiolres.931271 18198997

[B61] Gharib-Naseri K. Kheravii S. Keerqin C. Swick R. A. Choct M. Wu S. B. (2021). Differential expression of intestinal genes in necrotic enteritis challenged broiler chickens with 2 different *Clostridium perfringens* strains. Poult. Sci. 100, 100886. 10.1016/j.psj.2020.11.063 33516477 PMC7936145

[B62] Gibson P. R. Barrett J. S. (2010). The concept of small intestinal bacterial overgrowth in relation to functional gastrointestinal disorders. Nutrition 26, 1038–1043. 10.1016/j.nut.2010.01.005 20418060

[B63] Gill S. R. Pop M. Deboy R. T. Eckburg P. B. Turnbaugh P. J. Samuel B. S.. (2006). Metagenomic analysis of the human distal gut microbiome. Science 312, 1355–1359. 10.1126/science.1124234 16741115 PMC3027896

[B64] Guccione E. Leon-Kempis M. Pearson B. M. Hitchin E. Mulholland F. van Diemen P. M.. (2008). Amino acid-dependent growth of *Campylobacter jejuni*: key roles for aspartase (AspA) under microaerobic and oxygen-limited conditions and identification of AspB (Cj0762), essential for growth on glutamate. Mol. Microbiol. 69, 77–93. 10.1111/j.1365-2958.2008.06263.x 18433445

[B65] Hatano T. Saiki S. Okuzumi A. Mohney R. P. Hattori N. (2016). Identification of novel biomarkers for Parkinson's disease by metabolomic technologies. J. Neurol. Neurosurg. Psychiatry 87, 295–301. 10.1136/jnnp-2014-309676 25795009

[B66] Hayashi H. Takahashi R. Nishi T. Sakamoto M. Benno Y. (2005). Molecular analysis of jejunal, ileal, caecal and recto-sigut microbiotaoidal human colonic microbiota using 16S rRNA gene libraries and terminal restriction fragut microbiotaent length polymorphism. J. Med. Microbiol. 54, 1093–1101. 10.1099/jmm.0.45935-0 16192442

[B67] He L. Han M. Qiao S. He P. Li D. Li N.. (2015). Soybean antigen proteins and their intestinal sensitization activities. Curr. Protein Pept. Sci. 16, 613–621. 10.2174/1389203716666150630134602 26122781

[B68] Heintz-Buschart A. Pandey U. Wicke T. Sixel-Döring F. Janzen A. Sittig-Wiegand E.. (2018). The nasal and gut microbiome in Parkinson's disease and idiopathic rapid eye movement sleep behavior disorder. Mov. Disord. 33, 88–98. 10.1002/mds.27105 28843021 PMC5811909

[B69] Hendriks W. H. van Baal J. Bosch G. (2012). Ileal and faecal protein digestibility measurement in humans and other non-ruminants—a comparative species view. Br. J. Nutr. 108, S247–S257. 10.1017/S0007114512002395 23107535

[B70] Hernán M. A. Takkouche B. Caamaño-Isorna F. Gestal-Otero J. J. (2002). A meta-analysis of coffee drinking, cigarette smoking, and the risk of Parkinson's disease. Ann Neurol. 52, 276–284. 10.1002/ana.10277 12205639

[B71] Herrick M. K. Tansey M. G. (2021). Is LRRK2 the missing link between inflammatory bowel disease and Parkinson's disease? NPJ Parkinson's Dis. 7, 26. 10.1038/s41531-021-00170-1 33750819 PMC7943592

[B72] Hirayama M. Tsunoda M. Yamamoto M. Tsuda T. Ohno K. (2016). Serum tyrosine-to-phenylalanine ratio is low in Parkinson's disease. J. Parkinson's Dis. 6, 423–431. 10.3233/JPD-150736 27061063

[B73] Hou Y. He W. Hu S. Wu G. (2019). Composition of polyamines and amino acids in plant-source foods for human consumption. Amino Acids. 51, 1153–1165. 10.1007/s00726-019-02751-0 31197570

[B74] Hu X. Guo F. (2021). Amino acid sensing in metabolic homeostasis and health. Endocr Rev. 42, 56-76. 10.1210/endrev/bnaa026 33053153

[B75] Izco M. Vettorazzi A. de Toro M. Sáenz Y. Alvarez-Ervitim L. (2021). Oral sub-chronic ochratoxin A exposure induces gut microbiota alterations in mice. Toxins (Basel). 13, 106. 10.3390/toxins13020106 33535685 PMC7912851

[B76] Jiménez-Jiménez F. J. Alonso-Navarro H. García-Martín E. Agúndez J. (2020). Cerebrospinal and blood levels of amino acids as potential biomarkers for Parkinson's disease: review and meta-analysis. Eur. J. Neurol. 27, 2336–2347. 10.1111/ene.14470 32777152

[B77] Kang S. H. Lee J. Koh S. B. (2022). Constipation is associated with mild cognitive impairment in patients with de novo Parkinson's disease. J. Mov. Disord. 15, 38–42. 10.14802/jmd.21074 34781630 PMC8820884

[B78] Karkache I. Y. Damodaran J. R. Molstad D. Bradley E. W. (2021). Serine/threonine phosphatases in osteoclastogenesis and bone resorption. Gene 771, 145362. 10.1016/j.gene.2020.145362 33338510 PMC8204919

[B79] Kaye J. Gage H. Kimber A. Storey L. Trend P. (2006). Excess burden of constipation in Parkinson's disease: a pilot study. Mov. Disord. 21, 1270–1273. 10.1002/mds.20942 16700046

[B80] Kevin J. Portune M. B. Anne-Marie D. Daniel T. François B. Yolanda S. (2016). Gut microbiota role in dietary protein metabolism and health-related outcomes: the two sides of the coin. Trends Food Sci. Technol. 57, 213–232. 10.1016/j.tifs.2016.08.011

[B81] Killinger B. A. Madaj Z. Sikora J. W. Rey N. Haas A. J. Vepa Y. (2018). The vermiform appendix impacts the risk of developing Parkinson's disease. Sci. Transl. Med. 10, eaar5280. 10.1126/scitranslmed.aar5280 30381408 PMC6319259

[B82] Kim G. H. Lee Y. C. Kim T. J. Kim E. R. Hong S. N. Chang D. K.. (2022). Risk of neurodegenerative diseases in patients with inflammatory bowel disease: a nationwide population-based cohort study. J. Crohn's Colit. 16, 436–443. 10.1093/ecco-jcc/jjab162 34499125

[B83] Klingelhoefer L. Reichmann H. (2015). Pathogenesis of Parkinson disease—the gut-brain axis and environmental factors. Nat. Rev. Neurol. 11, 625–636. 10.1038/nrneurol.2015.197 26503923

[B84] Koh A. Molinaro A. Ståhlman M. Khan M. T. Schmidt C. Mannerås-Holm L.. (2018). Microbially produced imidazole propionate impairs insulin signaling through mTORC1. Cell 175, 947–961.e17. 10.1016/j.cell.2018.09.055 30401435

[B85] Krzystek-Korpacka M. Fleszar M. G. Bednarz-Misa I. Lewandowski Ł. Szczuka I. Kempiński R.. (2020). Transcriptional and metabolomic analysis of L-arginine/nitric oxide pathway in inflammatory bowel disease and its association with local inflammatory and angiogenic response: preliminary findings. Int. J. Mol. Sci. 21, 1641. 10.3390/ijms21051641 32121248 PMC7084352

[B86] Kubinak J. L. Round J. L. (2012). Toll-like receptors promote mutually beneficial commensal-host interactions. PLoS Pathog. 8, e1002785. 10.1371/journal.ppat.1002785 22910541 PMC3406078

[B87] Kuhn W. Roebroek R. Blom H. van Oppenraaij D. Przuntek H. Kretschmer A.. (1998). Elevated plasma levels of homocysteine in Parkinson's disease. Eur. Neurol. 40, 225–227. 10.1159/000007984 9813406

[B88] Kumari S. Kumaran S. S. Goyal V. Sharma R. K. Sinha N. Dwivedi S. N.. (2020). Identification of potential urine biomarkers in idiopathic parkinson's disease using NMR. Clin. Chim. Acta 510, 442–449. 10.1016/j.cca.2020.08.005 32791135

[B89] Lai S. W. Liao K. F. Lin C. L. Sung F. C. (2014). Irritable bowel syndrome correlates with increased risk of Parkinson's disease in Taiwan. Eur. J. Epidemiol. 29, 57–62. 10.1007/s10654-014-9878-3 24442494

[B90] Laparra J. M. Sanzm Y. (2010). Interactions of gut microbiota with functional food components and nutraceuticals. Pharmacol. Res. 61, 219–225. 10.1016/j.phrs.2009.11.001 19914380

[B91] Lee H. C. Wei Y. H. (2005). Mitochondrial biogenesis and mitochondrial DNA maintenance of mammalian cells under oxidative stress. Int. J. Biochem. Cell Biol. 37, 822–834. 10.1016/j.biocel.2004.09.010 15694841

[B92] Leikas J. V. Kohtala S. Theilmann W. Jalkanen A. J. Forsberg M. M. Rantamäki T. (2017). Brief isoflurane anesthesia regulates striatal AKT-GSK3β signaling and ameliorates motor deficits in a rat model of early-stage Parkinson's disease. J. Neurochem. 142, 456–463. 10.1111/jnc.14066 28488766 PMC5575520

[B93] Lewis K. Lutgendorff F. Phan V. Söderholm J. D. Sherman P. M. McKay D. M. (2010). Enhanced translocation of bacteria across metabolically stressed epithelia is reduced by butyrate. Inflamm. Bowel Dis. 16, 1138–1148. 10.1002/ibd.21177 20024905

[B94] Li M. Oshima T. Ito C. Yamada M. Tomita T. Fukui H.. (2021). Glutamine blocks interleukin-13-induced intestinal epithelial barrier dysfunction. Digestion 102, 170–179. 10.1159/000502953 31533100

[B95] Libao-Mercado A. J. Zhu C. L. Cant J. P. Lapierre H. Thibault J. N. Sève B.. (2009). Dietary and endogenous amino acids are the main contributors to microbial protein in the upper gut of normally nourished pigs. J. Nutr. 139, 1088–1094. 10.3945/jn.108.103267 19403708

[B96] Lin J. C. Lin C. S. Hsu C. W. Lin C. L. Kao C. H. (2016). Association between Parkinson's disease and inflammatory bowel disease: a nationwide Taiwanese retrospective cohort study. Inflamm. Bowel Dis. 22, 1049–1055. 10.1097/MIB.0000000000000735 26919462

[B97] Lin R. Liu W. Piao M. Zhu H. (2017). A review of the relationship between the gut microbiota and amino acid metabolism. Amino Acids 49, 2083–2090. 10.1007/s00726-017-2493-3 28932911

[B98] Lindahl M. Chalazonitis A. Palm E. Pakarinen E. Danilova T. Pham T. D.. (2020). Cerebral dopamine neurotrophic factor-deficiency leads to degeneration of enteric neurons and altered brain dopamine neuronal function in mice. Neurobiol. Dis. 134, 104696. 10.1016/j.nbd.2019.104696 31783118 PMC7000201

[B99] Liu B. Sjölander A. Pedersen N. L. Ludvigsson J. F. Chen H. Fang F.. (2021). Irritable bowel syndrome and Parkinson's disease risk: register-based studies. NPJ Parkinson's Dis. 7, 5. 10.1038/s41531-020-00145-8 33402695 PMC7785733

[B100] Liu J. R. Miao H. Deng D. Q. Vaziri N. D. Li P. Zhao Y. Y. (2021). Gut microbiota-derived tryptophan metabolism mediates renal fibrosis by aryl hydrocarbon receptor signaling activation. Cell. Mol. Life Sci. CMLS 78, 909–922. 10.1007/s00018-020-03645-1 32965514 PMC11073292

[B101] Liu S. Wang C. Chen Y. Peng S. Chen X. Tan Z. (2019). Association of SLC15A1 polymorphisms with susceptibility to dyslipidaemia in a Chinese Han population. J. Clin. Pharm. Therap. 44, 868–874. 10.1111/jcpt.13016 31454435

[B102] Lu S. Jiang H. Y. Shi Y. D. (2022). Association between irritable bowel syndrome and Parkinson's disease: a systematic review and meta-analysis. Acta Neurol. Scand. 145, 442–448. 10.1111/ane.13570 34908158

[B103] Luan H. Liu L. F. Tang Z. Zhang M. Chua K. K. Song J. X.. (2015). Comprehensive urinary metabolomic profiling and identification of potential noninvasive marker for idiopathic Parkinson's disease. Sci. Rep. 5, 13888. 10.1038/srep13888 26365159 PMC4568456

[B104] Lucas K. Maes M. (2013). Role of the toll like receptor (TLR) radical cycle in chronic inflammation: possible treatments targeting the TLR4 pathway. Mol. Neurobiol. 48, 190–204. 10.1007/s12035-013-8425-7 23436141 PMC7091222

[B105] Ma N. Ma X. (2019). Dietary amino acids and the gut-microbiome-immune axis: physiological metabolism and therapeutic prospects. Comprehens. Rev. Food Sci. Food Saf. 18, 221–242. 10.1111/1541-4337.12401 33337014

[B106] Ma X. Ma L. Wang Z. Liu Y. Long L. Ma X.. (2020). Clinical features and gut microbial alterations in anti-leucine-rich glioma-inactivated 1 encephalitis—a pilot study. Front. Neurol. 11, 585977. 10.3389/fneur.2020.585977 33193049 PMC7655127

[B107] Macfarlane G. T. Allison C. Gibson S. A. Cummings J. H. (1988). Contribution of the microflora to proteolysis in the human large intestine. J. Appl. Bacteriol. 64, 37–46. 10.1111/j.1365-2672.1988.tb02427.x 3127369

[B108] Macfarlane G. T. Macfarlane S. (2007). Models for intestinal fermentation: association between food components, delivery systems, bioavailability and functional interactions in the gut. Curr. Opin Biotechnol. 18, 156–162. 10.1016/j.copbio.2007.01.011 17276052

[B109] Macfarlane G. T. Macfarlane S. (2012). Bacteria, colonic fermentation, and gastrointestinal health. J. AOAC Int. 95, 50–60. 10.5740/jaoacint.SGE_Macfarlane 22468341

[B110] Machado S. Costa A. Pimentel B, F. Oliveira M. Alves R. C. (2020). A study on the protein fraction of coffee silverskin: protein/non-protein nitrogen and free and total amino acid profiles. Food Chem. 326, 126940. 10.1016/j.foodchem.2020.126940 32413751

[B111] Machiels K. Joossens M. Sabino J. De Preter V. Arijs I. Eeckhaut V.. (2014). A decrease of the butyrate-producing species *Roseburia hominis* and *Faecalibacterium prausnitzii* defines dysbiosis in patients with ulcerative colitis. Gut 63, 1275–1283. 10.1136/gutjnl-2013-304833 24021287

[B112] Mafra D. Barros A. F. Fouque D. (2013). Dietary protein metabolism by gut microbiota and its consequences for chronic kidney disease patients. Fut. Microbiol. 8, 1317–1323. 10.2217/fmb.13.103 24059921

[B113] Maini Rekdal V. Bess E. N. Bisanz J. E. Turnbaugh P. J. Balskus E. P. (2019). Discovery and inhibition of an interspecies gut bacterial pathway for Levodopa metabolism. Science (New York, N.Y.) 364, eaau6323. 10.1126/science.aau6323 31196984 PMC7745125

[B114] Malek N. (2019). Deep brain stimulation in Parkinson's disease. Neurol. India 67, 968–978. 10.4103/0028-3886.266268 31512617

[B115] Manasanch E. E. Orlowski R. Z. (2017). Proteasome inhibitors in cancer therapy. Nat. Rev. Clin. Oncol. 14, 417–433. 10.1038/nrclinonc.2016.206 28117417 PMC5828026

[B116] Manole E. Dumitrescu L. Niculite C. Popescu B. O. Ceafalan L. C. (2021). Potential roles of functional bacterial amyloid proteins, bacterial biosurfactants and other putative gut microbiota products in the etiopathogeny of Parkinson's disease. Biocell 45, 1–16. 10.32604/biocell.2021.013452

[B117] Marrinan S. Emmanuel A. V. Burn D. J. (2014). Delayed gastric emptying in Parkinson's disease. Mov. Disord. 29, 23–32. 10.1002/mds.25708 24151126

[B118] Martí I Líndez A. A. Reith W. (2021). Arginine-dependent immune responses. Cell. Mol. Life Sci. CMLS 78, 5303–5324. 10.1007/s00018-021-03828-4 34037806 PMC8257534

[B119] Mayne K. White J. A. McMurran C. E. Rivera F. J. de la Fuente A. G. (2020). Aging and neurodegenerative disease: is the adaptive immune system a friend or foe? Front. Aging Neurosci. 12, 572090. 10.3389/fnagi.2020.572090 33173502 PMC7538701

[B120] Mertsalmi T. H. Aho V. Pereira P. Paulin L. Pekkonen E. Auvinen P.. (2017). More than constipation—bowel symptoms in Parkinson's disease and their connection to gut microbiota. Eur. J. Neurol. 24, 1375–1383. 10.1111/ene.13398 28891262

[B121] Mertsalmi T. H. But A. Pekkonen E. Scheperjans F. (2021). Irritable bowel syndrome and risk of Parkinson's disease in finland: a nationwide registry-based cohort study. J. Parkinson's Dis. 11, 641–651. 10.3233/JPD-202330 33646176 PMC8150653

[B122] Metges C. C. (2000). Contribution of microbial amino acids to amino acid homeostasis of the host. J Nutr. 130, 1857S−1864S. 10.1093/jn/130.7.1857S 10867063

[B123] Metges C. C. El-Khoury A. E. Henneman L. Petzke K. J. Grant I. Bedri S.. (1999). Availability of intestinal microbial lysine for whole body lysine homeostasis in human subjects. Am. J. Physiol. 277, E597–E607. 10.1152/ajpendo.1999.277.4.E597 10516118

[B124] Metges C. C. Petzke K. J. Hennig U. (1996). Gas chromatography/combustion/isotope ratio mass spectrometric comparison of N-acetyl- and N-pivaloyl amino acid esters to measure 15N isotopic abundances in physiological samples: a pilot study on amino acid synthesis in the upper gastro-intestinal tract of minipigs. J. Mass Spectr. JMS 31, 367–376. 10.1002/(SICI)1096-9888(199604)31:4<367::AID-JMS310>3.0.CO;2-V 8799283

[B125] Mu C. Yang Y. Yu K. Yu M. Zhang C. Su Y.. (2017). Alteration of metabolomic markers of amino-acid metabolism in piglets with in-feed antibiotics. Amino Acids 49, 771–781. 10.1007/s00726-017-2379-4 28101652

[B126] Müller T. Trommer I. Muhlack S. Mueller B. K. (2016). Levodopa increases oxidative stress and repulsive guidance molecule A levels: a pilot study in patients with Parkinson's disease. J. Neural Transm. 123, 401–406. 10.1007/s00702-016-1519-4 26880022

[B127] Nagaraja S. Cai M. W. Sun J. Varet H. Sarid L. Trebicz-Geffen M.. (2021). Queuine is a nutritional regulator of *Entamoeba histolytica* response to oxidative stress and a virulence attenuator. MBio. 12, e03549–e03520. 10.1128/mBio.03549-20 33688012 PMC8092309

[B128] Navarro A. Boveris A. (2009). Brain mitochondrial dysfunction and oxidative damage in Parkinson's disease. J. Bioenerg. Biomembr. 41, 517–521. 10.1007/s10863-009-9250-6 19915964

[B129] Neis E. P. Dejong C. H. Rensen S. S. (2015). The role of microbial amino acid metabolism in host metabolism. Nutrients 7, 2930–2946. 10.3390/nu7042930 25894657 PMC4425181

[B130] Nielsen P. M. Mariager C. Ø. Mølmer M. Sparding N. Genovese F. Karsdal M. A.. (2020). Hyperpolarized [1-13 C] alanine production: a novel imaging biomarker of renal fibrosis. Magn. Reson. Med. 84, 2063–2073. 10.1002/mrm.28326 32452096

[B131] Niu X. L. Liu L. Song Z. X. Li Q. Wang Z. H. Zhang J. L.. (2016). Prevalence of small intestinal bacterial overgrowth in Chinese patients with Parkinson's disease. J. Neural Transm. (Vienna, Austria: 1996) 123, 1381–1386. 10.1007/s00702-016-1612-8 27589873

[B132] Noh H. Jang J. Kwon S. Cho S. Y. Jung W. S. Moon S. K.. (2020). The impact of korean medicine treatment on the incidence of Parkinson's disease in patients with inflammatory bowel disease: a nationwide population-based cohort study in South Korea. J. Clin. Med. 9, 2422. 10.3390/jcm9082422 32731605 PMC7463832

[B133] Ohman L. Simrén M. (2010). Pathogenesis of IBS: role of inflammation, immunity and neuroimmune interactions. Nat. Rev. Gastroenterol. Hepatol. 7, 163–173. 10.1038/nrgastro.2010.4 20101257

[B134] Park S. Kim J. Chun J. Han K. Soh H. Kang E. A.. (2019). Patients with inflammatory bowel disease are at an increased risk of Parkinson's disease: a South Korean nationwide population-based study. J. Clin. Med. 8, 1191. 10.3390/jcm8081191 31398905 PMC6723604

[B135] Peng L. Li Z. R. Green R. S. Holzman I. R. Lin J. (2009). Butyrate enhances the intestinal barrier by facilitating tight junction assembly via activation of AMP-activated protein kinase in Caco-2 cell monolayers. J. Nutr. 139, 1619–1625. 10.3945/jn.109.104638 19625695 PMC2728689

[B136] Peter I. Dubinsky M. Bressman S. Park A. Lu C. Chen N. (2018). Anti-tumor necrosis factor therapy and incidence of Parkinson disease among patients with inflammatory bowel disease. JAMA Neurol. 75, 939–946. 10.1001/jamaneurol.2018.0605 29710331 PMC6142934

[B137] Postuma R. B. (2015). Can Parkinson's disease come from the gut? Mov. Disord. 30, 1325. 10.1002/mds.26337 26227405

[B138] Potrykus J. White R. L. Bearne S. L. (2008). Proteomic investigation of amino acid catabolism in the indigenous gut anaerobe *Fusobacterium varium*. Proteomics 8, 2691–2703. 10.1002/pmic.200700437 18546150

[B139] Raskov H. Burcharth J. Pommergaard H. C. Rosenberg J. (2016). Irritable bowel syndrome, the microbiota and the gut-brain axis. Gut Microbes 7, 365–383. 10.1080/19490976.2016.1218585 27472486 PMC5046167

[B140] Raval U. Harary J. M. Zeng E. Pasinetti G. M. (2020). The dichotomous role of the gut microbiome in exacerbating and ameliorating neurodegenerative disorders. Expert Rev. Neurother. 20, 673–686. 10.1080/14737175.2020.1775585 32459513 PMC7387222

[B141] Rhee S. H. (2014). Lipopolysaccharide: basic biochemistry, intracellular signaling, and physiological impacts in the gut. Intest Res. 12, 90–95. 10.5217/ir.2014.12.2.90 25349574 PMC4204704

[B142] Rist V. T. Weiss E. Eklund M. Mosenthin R. (2013). Impact of dietary protein on microbiota composition and activity in the gastrointestinal tract of piglets in relation to gut health: a review. Animal 7, 1067–1078. 10.1017/S1751731113000062 23410993

[B143] Roager H. M. Licht T. R. (2018). Microbial tryptophan catabolites in health and disease. Nat. Commun. 9, 3294. 10.1038/s41467-018-05470-4 30120222 PMC6098093

[B144] Rom O. Liu Y. Liu Z. Zhao Y. Wu J. Ghrayeb A.. (2020). Glycine-based treatment ameliorates NAFLD by modulating fatty acid oxidation, glutathione synthesis, and the gut microbiome. Sci. Transl. Med. 12, eaaz2841. 10.1126/scitranslmed.aaz2841 33268508 PMC7982985

[B145] Rosales C. Demaurex N. Lowell C. A. Uribe-Querol E. (2016). Neutrophils: their role in innate and adaptive immunity. J. Immunol. Res. 2016, 1469780. 10.1155/2016/1469780 27006954 PMC4783580

[B146] Rychlik J. L. Russell J. B. (2002). The adaptation and resistance of *Clostridium aminophilum* F to the butyrivibriocin-like substance of *Butyrivibrio fibrisolvens* JL5 and monensin. FEMS Microbiol. Lett. 209, 93–98. 10.1111/j.1574-6968.2002.tb11115.x 12007660

[B147] Sampson T. R. Debelius J. W. Thron T. Janssen S. Shastri G. G. Ilhan Z. E. (2016). Gut microbiota regulate motor deficits and neuroinflammation in a model of Parkinson's disease. Cell 167, 1469–1480.e12. 10.1016/j.cell.2016.11.018 27912057 PMC5718049

[B148] Sampson T. R. Mazmanian S. K. (2015). Control of brain development, function, and behavior by the microbiome. Cell Host Microbe 17, 565–576. 10.1016/j.chom.2015.04.011 25974299 PMC4442490

[B149] Sánchez-Andrea I. Guedes I. A. Hornung B. Boeren S. Lawson C. E. Sousa D. Z.. (2020). The reductive glycine pathway allows autotrophic growth of *Desulfovibrio desulfuricans*. Nat. Commun. 11, 5090. 10.1038/s41467-020-18906-7 33037220 PMC7547702

[B150] Sankarasubramanian J. Ahmad R. Avuthu N. Singh A. B. Guda C. (2020). Gut microbiota and metabolic specificity in ulcerative colitis and Crohn's disease. Front. Med. 7, 606298. 10.3389/fmed.2020.606298 33330572 PMC7729129

[B151] Santos García D. García Roca L. de Deus Fonticoba T. Cores Bartolomé C. Naya Ríos L. Canfield H.. (2022). Constipation predicts cognitive decline in Parkinson's disease: results from the COPPADIS cohort at 2-year follow-up and comparison with a control group. J. Parkinson's Dis. 12, 315–331. 10.3233/JPD-212868 34602501

[B152] Sarkar S. Gough B. Raymick J. Beaudoin M. A. Ali S. F. Virmani A.. (2015). Histopathological and electrophysiological indices of rotenone-evoked dopaminergic toxicity: Neuroprotective effects of acetyl-L-carnitine. Neurosci. Lett. 606, 53–59. 10.1016/j.neulet.2015.08.044 26321151

[B153] Sarkar S. Raymick J. Imam S. (2016). Neuroprotective and therapeutic strategies against parkinson's disease: recent perspectives. Int. J. Mol. Sci. 17, 904. 10.3390/ijms17060904 27338353 PMC4926438

[B154] Savica R. Carlin J. M. Grossardt B. R. Bower J. H. Ahlskog J. E. Maraganore D. M.. (2009). Medical records documentation of constipation preceding Parkinson disease: a case-control study. Neurology 73, 1752–1758. 10.1212/WNL.0b013e3181c34af5 19933976 PMC2788809

[B155] Scheperjans F. Aho V. Pereira P. A. Koskinen K. Paulin L. Pekkonen E.. (2015). Gut microbiota are related to Parkinson's disease and clinical phenotype. Mov. Disord. 30,350–358. 10.1002/mds.26069 25476529

[B156] Schernhammer E. Chen H. Ritz B. (2006). Circulating melatonin levels: possible link between Parkinson's disease and cancer risk? Cancer Causes Control 17, 577–582. 10.1007/s10552-005-9002-9 16596313

[B157] Schierack P. Walk N. Reiter K. Weyrauch K. D. Wieler L. H. (2007). Composition of intestinal Enterobacteriaceae populations of healthy domestic pigs. Microbiology(Reading, England) 153, 3830–3837. 10.1099/mic.0.2007/010173-0 17975092

[B158] Schoultz I. Keita Å. V. (2019). The intestinal barrier and current techniques for the assessment of gut permeability. Cells 8, 193. 10.3390/cells8020193 32824536 PMC7463717

[B159] Schoultz I. Keita Å. V. (2020). The intestinal barrier and current techniques for the assessment of gut permeability. Cells 9, 1909. 10.3390/cells9081909 32824536 PMC7463717

[B160] Schwiertz A. Spiegel J. Dillmann U. Grundmann D. Bürmann J. Faßbender K.. (2018). Fecal markers of intestinal inflammation and intestinal permeability are elevated in Parkinson's disease. Parkinsonism Relat. Disord. 50, 104–107. 10.1016/j.parkreldis.2018.02.022 29454662

[B161] Shao Y. Li T. Liu Z. Wang X. Xu X. Li S.. (2021). Comprehensive metabolic profiling of Parkinson's disease by liquid chromatography-mass spectrometry. Mol. Neurodegener. 16, 4. 10.1186/s13024-021-00425-8 33485385 PMC7825156

[B162] Shen G. Wu J. Ye B. C. Qi N. (2021). Gut microbiota-derived metabolites in the development of diseases. Can. J. Infect. Dis. Med. Microbiol. 2021, 6658674. 10.1155/2021/6658674 33505541 PMC7815404

[B163] Sherman M. Y. Goldberg A. L. (2001). Cellular defenses against unfolded proteins: a cell biologist thinks about neurodegenerative diseases. Neuron 29, 15–32. 10.1016/S0896-6273(01)00177-5 11182078

[B164] Sokol H. Pigneur B. Watterlot L. Lakhdari O. Bermúdez-Humarán L. G. Gratadoux J. J.. (2008). Faecalibacterium prausnitzii is an anti-inflammatory commensal bacterium identified by gut microbiota analysis of Crohn disease patients. Proc. Natl. Acad. Sci. USA. 105, 16731–16736. 10.1073/pnas.0804812105 18936492 PMC2575488

[B165] Sonnenburg E. D. Sonnenburg J. L. (2014). Starving our microbial self: the deleterious consequences of a diet deficient in microbiota-accessible carbohydrates. Cell Metab. 20, 779–786. 10.1016/j.cmet.2014.07.003 25156449 PMC4896489

[B166] Stokholm M. G. Danielsen E. H. Hamilton-Dutoit S. J. Borghammer P. (2016). Pathological α-synuclein in gastrointestinal tissues from prodromal Parkinson disease patients. Ann. Neurol. 79, 940–949. 10.1002/ana.24648 27015771

[B167] Sugihara P. T. Sutter V. L. Attebery H. R. Bricknell K. S. Finegold S. M. (1974). Isolation of *Acidaminococcus fermentans* and *Megasphaera elsdenii* from normal human feces. Appl. Microbiol. 27, 274–275. 10.1128/am.27.1.274-275.1974 4589136 PMC380007

[B168] Sulzer D. (2007). Multiple hit hypotheses for dopamine neuron loss in Parkinson's disease. Trends Neurosci. 30, 244–250. 10.1016/j.tins.2007.03.009 17418429

[B169] Sun L. Liu Q. Bao C. Fan J. (2017). Comparison of free total amino acid compositions and their functional classifications in 13 wild edible mushrooms. Molecules (Basel, Switzerland) 22, 350. 10.3390/molecules22030350 28245582 PMC6155212

[B170] Sun M. F. Shen Y. Q. (2018). Dysbiosis of gut microbiota and microbial metabolites in Parkinson's Disease. Ageing Res. Rev. 45, 53–61. 10.1016/j.arr.2018.04.004 29705121

[B171] Svensson E. Henderson V. W. Borghammer P. Horváth-Puhó E. Sørensen H. T. (2016). Constipation and risk of Parkinson's disease: a Danish population-based cohort study. Parkinsonism Relat Disord. 28, 18–22. 10.1016/j.parkreldis.2016.05.016 27234704

[B172] Sylte M. J. Shippy D. C. Bearson B. L. Bearson S. (2020). Detection of *Campylobacter jejuni* liver dissemination in experimentally colonized turkey poults. Poult. Sci. 99, 4028–4033. 10.1016/j.psj.2020.03.042 32731990 PMC7597910

[B173] Tabler T. W. Greene E. S. Orlowski S. K. Hiltz J. Z. Anthony N. B. Dridi S. (2020). Intestinal barrier integrity in heat-stressed modern broilers and their ancestor wild jungle fowl. Front. Vet. Sci. 7, 249. 10.3389/fvets.2020.00249 32457922 PMC7220999

[B174] Tambasco N. Romoli M. Calabresi P. (2018). Levodopa in Parkinson's disease: current status and future developments. Curr. Neuropharmacol. 16, 1239–1252. 10.2174/1570159X15666170510143821 28494719 PMC6187751

[B175] Tan A. H. Mahadeva S. Thalha A. M. Gibson P. R. Kiew C. K. Yeat C. M.. (2014). Small intestinal bacterial overgrowth in Parkinson's disease. Parkinsonism Relat. Disord. 20, 535–540. 10.1016/j.parkreldis.2014.02.019 24637123

[B176] Thalacker-Mercer A. Riddle E. Barre L. (2020). Protein and amino acids for skeletal muscle health in aging. Adv. Food Nutr. Res. 91, 29–64. 10.1016/bs.afnr.2019.08.002 32035599

[B177] Toczylowska B. Zieminska E. Michałowska M. Chalimoniuk M. Fiszer U. (2020). Changes in the metabolic profiles of the serum and putamen in Parkinson's disease patients—in vitro and in vivo NMR spectroscopy studies. Brain Res. 1748, 147118. 10.1016/j.brainres.2020.147118 32931820

[B178] Torrallardona D. Harris C. I. Coates M. E. Fuller M. F. (1996). Microbial amino acid synthesis and utilization in rats: incorporation of 15N from 15NH4Cl into lysine in the tissues of germ-free and conventional rats. Br. J. Nutr. 76, 689–700. 10.1079/BJN19960076 8958003

[B179] Trist B. G. Hare D. J. Double K. L. (2019). Oxidative stress in the aging substantia nigra and the etiology of Parkinson's disease. Aging Cell 18, e13031. 10.1111/acel.13031 31432604 PMC6826160

[B180] van de Wouw M. Schellekens H. Dinan T. G. Cryan J. F. (2017). Microbiota-gut-brain axis: modulator of host metabolism and appetite. J. Nutr. 147, 727–745. 10.3945/jn.116.240481 28356427

[B181] van Kessel S. P. Frye A. K. El-Gendy A. O. Castejon M. Keshavarzian A. van Dijk G.. (2019). Gut bacterial tyrosine decarboxylases restrict levels of levodopa in the treatment of Parkinson's disease. Nat. Commun. 10, 310. 10.1038/s41467-019-08294-y 30659181 PMC6338741

[B182] Vascellari S. Palmas V. Melis M. Pisanu S. Cusano R. Uva P.. (2020). Gut microbiota and metabolome alterations associated with Parkinson's disease. mSystems 5, e00561–e00520. 10.1128/mSystems.00561-20 32934117 PMC7498685

[B183] Venkatesh M. Mukherjee S. Wang H. Li H. Sun K. Benechet A. P.. (2014). Symbiotic bacterial metabolites regulate gastrointestinal barrier function via the xenobiotic sensor PXR and toll-like receptor 4. Immunity 41, 296–310. 10.1016/j.immuni.2014.06.014 25065623 PMC4142105

[B184] Verzola D. Picciotto D. Saio M. Aimasso F. Bruzzone F. Sukkar S. G.. (2020). Low protein diets and plant-based low protein diets: do they meet protein requirements of patients with chronic kidney disease? Nutrients 13, 83. 10.3390/nu13010083 33383799 PMC7824653

[B185] Villumsen M. Aznar S. Pakkenberg B. Jess T. Brudek T. (2019). Inflammatory bowel disease increases the risk of Parkinson's disease: a Danish nationwide cohort study 1977-2014. Gut 68, 18–24. 10.1136/gutjnl-2017-315666 29785965

[B186] Wallace K. L. Zheng L. B. Kanazawa Y. Shih D. Q. (2014). Immunopathology of inflammatory bowel disease. World J. Gastroenterol. 20, 6–21. 10.3748/wjg.v20.i1.6 24415853 PMC3886033

[B187] Wan J. Liu H. Chu J. Zhang H. (2019). Functions and mechanisms of lysine crotonylation. J. Cell. Mol. Med. 23, 7163–7169. 10.1111/jcmm.14650 31475443 PMC6815811

[B188] Wang Q. Holst J. (2015). L-type amino acid transport and cancer: targeting the mTORC1 pathway to inhibit neoplasia. Am. J. Cancer Res. 5, 1281–1294. 26101697 PMC4473310

[B189] Wang S. L. Shao B. Z. Zhao S. B. Fang J. Gu L. Miao C. Y.. (2018). Impact of paneth cell autophagy on inflammatory bowel disease. Front. Immunol. 9, 693. 10.3389/fimmu.2018.00693 29675025 PMC5895641

[B190] Weiner WJ. (2002). An algorithm (decision tree) for the management of Parkinson's disease (2001): treatment guidelines. Neurology 58, 156. 10.1212/WNL.58.1.156 11791587

[B191] Wells J. M. Rossi O. Meijerink M. van Baarlen P. (2011). Epithelial crosstalk at the microbiota-mucosal interface. Proc. Natl. Acad. Sci. USA. 108 (Suppl 1), 4607–4614. 10.1073/pnas.1000092107 20826446 PMC3063605

[B192] Whitehead T. R. Cotta M. A. (2004). Isolation and identification of hyper-ammonia producing bacteria from swine manure storage pits. Curr. Microbiol. 48, 20–26. 10.1007/s00284-003-4084-7 15018098

[B193] Wolters E. Braak H. (2006). Parkinson's disease: premotor clinico-pathological correlations. J. Neural Transm. Supplementum (70), 309–319. 10.1007/978-3-211-45295-0_47 17017546

[B194] Wu L. Deng H. (2020). Defluorination of 4-fluorothreonine by threonine deaminase. Organ. Biomol. Chem. 18, 6236–6240. 10.1039/D0OB01358G 32729605

[B195] Wu L. Tang Z. Chen H. Ren Z. Ding Q. Liang K.. (2021). Mutual interaction between gut microbiota and protein/amino acid metabolism for host mucosal immunity and health. Anim. Nutr. 7, 11–16. 10.1016/j.aninu.2020.11.003 33997326 PMC8110859

[B196] Xie X. Luo X. Xie M. (2017). Association between Parkinson's disease and risk of colorectal cancer. Parkinsonism Relat. Disord. 35, 42–47. 10.1016/j.parkreldis.2016.11.011 27913126

[B197] Yang D. Zhao D. Ali Shah S. Z. Wu W. Lai M. Zhang X.. (2019). The role of the gut microbiota in the pathogenesis of Parkinson's disease. Front. Neurol. 10, 1155. 10.3389/fneur.2019.01155 31781020 PMC6851172

[B198] Yang K. (2022). Regulation of treg cell metabolism and function in non-lymphoid tissues. Front. Immunol. 13, 909705. 10.3389/fimmu.2022.909705 35720275 PMC9200993

[B199] Yoon S. Y. Shin J. Heo S. J. Chang J. S. Sunwoo M. K. Kim Y. W. (2022). Irritable bowel syndrome and subsequent risk of Parkinson's disease: a nationwide population-based matched-cohort study. J. Neurol. 269, 1404–1412. 10.1007/s00415-021-10688-2 34255181

[B200] Yuan Y. S. Zhou X. J. Tong Q. Zhang L. Zhang L. Qi Z. Q.. (2013). Change in plasma levels of amino acid neurotransmitters and its correlation with clinical heterogeneity in early Parkinson's disease patients. CNS Neurosci. Therap. 19, 889–896. 10.1111/cns.12165 23981689 PMC6493594

[B201] Zhang J. Zhu S. Ma N. Johnston L. J. Wu C. Ma X. (2021). Metabolites of microbiota response to tryptophan and intestinal mucosal immunity: A therapeutic target to control intestinal inflammation. Med. Res. Rev. 41, 1061–1088. 10.1002/med.21752 33174230

[B202] Zhang X. Svn Z. Liv M. Yang Y. Zeng R. Huang Q.. (2021). Association between irritable bowel syndrome and risk of Parkinson's disease: a systematic review and meta-analysis. Front. Neurol. 12, 720958. 10.3389/fneur.2021.720958 34630293 PMC8492947

[B203] Zhang Y. He X. Qian Y. Xu S. Mo C. Yan Z.. (2022). Plasma branched-chain and aromatic amino acids correlate with the gut microbiota and severity of Parkinson's disease. NPJ Parkinson's Dis. 8, 48. 10.1038/s41531-022-00312-z 35449203 PMC9023571

[B204] Zhao J. Zhang X. Liu H. Brown M. A. Qiao S. (2019). Dietary protein and gut microbiota composition and function. Curr. Protein Peptide Sci. 20, 145–154. 10.2174/1389203719666180514145437 29756574

[B205] Zhao Z. Liu W. (2020). Pancreatic cancer: a review of risk factors, diagnosis, and treatment. Technol. Cancer Res. Treatm. 19, 1533033820962117. 10.1177/1533033820962117 33357065 PMC7768873

[B206] Zheng J. H. Sun W. H. Ma J. J. Wang Z. D. Chang Q. Q. Dong L. R.. (2022). Resting-state functional magnetic resonance imaging in patients with Parkinson's disease with and without constipation: a prospective study. Clin. Auton. Res. 32, 51–58. 10.1007/s10286-022-00851-8 35059875

[B207] Zhou Y. Su Y. Xu W. Wang W. Yao S. (2019). Constipation increases disability and decreases dopamine levels in the nigrostriatal system through gastric inflammatory factors in Parkinson's disease. Curr. Neurovasc. Res. 16, 241–249. 10.2174/1567202616666190618170103 31258082

[B208] Zhu F. Li C. Gong J. Zhu W. Gu L. Li N. (2019). The risk of Parkinson's disease in inflammatory bowel disease: a systematic review and meta-analysis. Dig. Liver Dis. 51, 38–42. 10.1016/j.dld.2018.09.017 30309751

[B209] Zhu Y. Yuan M. Liu Y. Yang F. Chen W. Z. Xu Z. Z.. (2022). Association between inflammatory bowel diseases and Parkinson's disease: systematic review and meta-analysis. Neural Regener. Res. 17, 344–353. 10.4103/1673-5374.317981 34269209 PMC8463981

[B210] Zou B. Sun Y. Xu Z. Chen Y. Li L. Lin L.. (2021). Rapid simultaneous determination of gut microbial phenylalanine, tyrosine, and tryptophan metabolites in rat serum, urine, and faeces using LC-MS/MS and its application to a type 2 diabetes mellitus study. Biomed. Chromatogr. BMC 35, e4985. 10.1002/bmc.4985 33200425

